# The zebrafish as a novel model for the *in vivo* study of *Toxoplasma gondii* replication and interaction with macrophages

**DOI:** 10.1242/dmm.043091

**Published:** 2020-07-20

**Authors:** Nagisa Yoshida, Marie-Charlotte Domart, Christopher J. Peddie, Artur Yakimovich, Maria J. Mazon-Moya, Thomas A. Hawkins, Lucy Collinson, Jason Mercer, Eva-Maria Frickel, Serge Mostowy

**Affiliations:** 1 Host-Toxoplasma Interaction Laboratory, The Francis Crick Institute, 1 Midland Road, London NW1 1BF, UK; 2 Section of Microbiology, MRC Centre for Molecular Bacteriology and Infection, Imperial College London, London SW7 2AZ, UK; 3 Department of Infection Biology, London School of Hygiene and Tropical Medicine, Keppel Street, London WC1E 7HT, UK; 4 Electron Microscopy Science Technology Platform, The Francis Crick Institute, 1 Midland Road, London NW1 1BF, UK; 5 MRC-Laboratory for Molecular Cell Biology, University College London, Gower Street, London, WC1E 6BT, UK; 6 Artificial Intelligence for Life Sciences CIC, 40 Gowers Walk, London, E1 8BH, UK; 7 Department of Cell and Developmental Biology, University College London, Gower Street, London, WC1E 6BT, UK; 8 Institute of Microbiology and Infection, School of Biosciences, University of Birmingham, Edgbaston, B15 2TT, UK

**Keywords:** CLEM, *In vivo*, Macrophages, *Toxoplasma gondii*, Zebrafish

## Abstract

*Toxoplasma gondii* is an obligate intracellular parasite capable of invading any nucleated cell. Three main clonal lineages (type I, II, III) exist and murine models have driven the understanding of general and strain-specific immune mechanisms underlying *Toxoplasma* infection. However, murine models are limited for studying parasite-leukocyte interactions *in vivo*, and discrepancies exist between cellular immune responses observed in mouse versus human cells. Here, we developed a zebrafish infection model to study the innate immune response to *Toxoplasma in vivo*. By infecting the zebrafish hindbrain ventricle, and using high-resolution microscopy techniques coupled with computer vision-driven automated image analysis, we reveal that *Toxoplasma* invades brain cells and replicates inside a parasitophorous vacuole to which type I and III parasites recruit host cell mitochondria. We also show that type II and III strains maintain a higher infectious burden than type I strains. To understand how parasites are cleared *in vivo*, we further analyzed *Toxoplasma-*macrophage interactions using time-lapse microscopy and three-dimensional correlative light and electron microscopy (3D CLEM). Time-lapse microscopy revealed that macrophages are recruited to the infection site and play a key role in *Toxoplasma* control. High-resolution 3D CLEM revealed parasitophorous vacuole breakage in brain cells and macrophages *in vivo*, suggesting that cell-intrinsic mechanisms may be used to destroy the intracellular niche of tachyzoites*.* Together, our results demonstrate *in vivo* control of *Toxoplasma* by macrophages, and highlight the possibility that zebrafish may be further exploited as a novel model system for discoveries within the field of parasite immunity.

This article has an associated First Person interview with the first author of the paper.

## Abstract (precis)

**Summary:** We have established a novel zebrafish infection model to investigate *Toxoplasma* replication *in vivo*, and demonstrate a key role for macrophages in parasite control.

## INTRODUCTION

*Toxoplasma gondii* is a successful human pathogen that often remains asymptomatic, although complications can arise in the immunocompromised and in neonates if infection is contracted during pregnancy (Pappas et al., 2009). *Toxoplasma* exist as invasive rapidly replicating tachyzoites in intermediate hosts (such as rodents and livestock), and convert into bradyzoite cysts in immune-privileged sites and long-lived cells (such as the brain and muscle tissue) during chronic infection (Pittman and Knoll, 2015). Once inside the host cell, parasites reside in a non-fusogenic parasitophorous vacuole (PV), where *Toxoplasma* asexually replicates (Clough and Frickel, 2017). Egress leads to dissemination into neighboring tissues, culminating in systemic infection. Predation of intermediate hosts by the definitive feline host completes the *Toxoplasma* life cycle. Control of infection by the host immune response is thus critical for host survival and for continued parasite transmission. As a result of its well-understood life cycle, *Toxoplasma* has emerged as a valuable model organism to understand the balance between pathogen survival and innate cellular immune control.

Three clonal lineages of *Toxoplasma* dominate across Europe and South America; namely, the type I, II and III strains (Howe and Sibley, 1995). These three closely related *Toxoplasma* strains have been characterized by the severity of infections they cause in murine models (Gazzinelli et al., 2014). Infection with type I parasites causes acute mouse mortality, whereas infection with type II and type III parasites progresses towards chronic infection (Saeij et al., 2005; Szabo and Finney, 2017). In humans, it is thought that type II strains predominate in Europe, yet strain-dependent differences in pathogenesis and host responses are poorly understood (Ajzenberg et al., 2002, 2009).

Innate immune mechanisms against *Toxoplasma* infection have been studied *in vitro* using both murine and human cell lines, and *in vivo* using mice. *In vivo* studies have shown that monocytes and neutrophils are recruited to the intestine upon oral infection, and are the major cell types infected with *Toxoplasma* both *in vivo* and *ex vivo* in human peripheral blood (Channon et al., 2000; Gregg et al., 2013; Coombes et al., 2013; Harker et al., 2015). The importance of neutrophils in parasite control *in vivo* is not fully understood, although neutrophil-specific depletion studies have suggested a minor protective role against *Toxoplasma* (Del Rio et al., 2001; Denkers et al., 2012). In contrast, inflammatory monocytes are the first responders to infection and are crucial for controlling acute *Toxoplasma* infection (Mordue and Sibley, 2003; Robben et al., 2005; Dunay et al., 2008). Pioneering work identified the ability of macrophages to kill *Toxoplasma* (Murray et al., 1979; Murray and Cohn, 1979), by employing both IFN-γ-dependent and -independent mechanisms to control intracellular parasite replication (Sibley et al., 1991; Andrade et al., 2005; Saeij and Frickel, 2017).

While the mouse is a natural intermediate host and remains an important model to understand *Toxoplasma* pathogenesis, differences are emerging between the mouse and human in mechanisms of parasite control (Gazzinelli et al., 2014; Yarovinsky et al., 2008; Haldar et al., 2015; Tosh et al., 2016; Sher et al., 2017; Safronova et al., 2019). Therefore, to complement *in vivo* murine studies, a novel animal model can benefit analysis of *Toxoplasma* control on a cellular and molecular level. Zebrafish are a well-established model for studying infection and immunity (Renshaw and Trede, 2012; Yoshida et al., 2017; Torraca and Mostowy, 2018; Gomes and Mostowy, 2020). Coupled with their optical accessibility during early development, zebrafish larvae are highly suited for non-invasive study of *Toxoplasma* infection and host response in real time *in vivo* (Torraca and Mostowy, 2018; Gomes and Mostowy, 2020).

Here, we developed a zebrafish infection model to study strain-dependent infectivity and leukocyte response to *Toxoplasma* infection in the hindbrain. We show that *Toxoplasma* invade and replicate inside brain cells including post-mitotic neurons, and that type II (Pru) and III (CEP) parasites maintain a higher infectious burden than type I (RH) parasites. We also demonstrate that macrophages are crucial in the clearance of viable parasites, and use high-resolution three-dimensional correlative light and electron microscopy (3D CLEM) techniques to reveal a discontinuous PV in brain cells and macrophages. Our zebrafish infection model can therefore be used as a novel platform to enable unprecedented discoveries in strain-dependent parasite immunity.

## RESULTS

### Intracellular *Toxoplasma* replicate in the zebrafish hindbrain ventricle

**Fig. 1.**
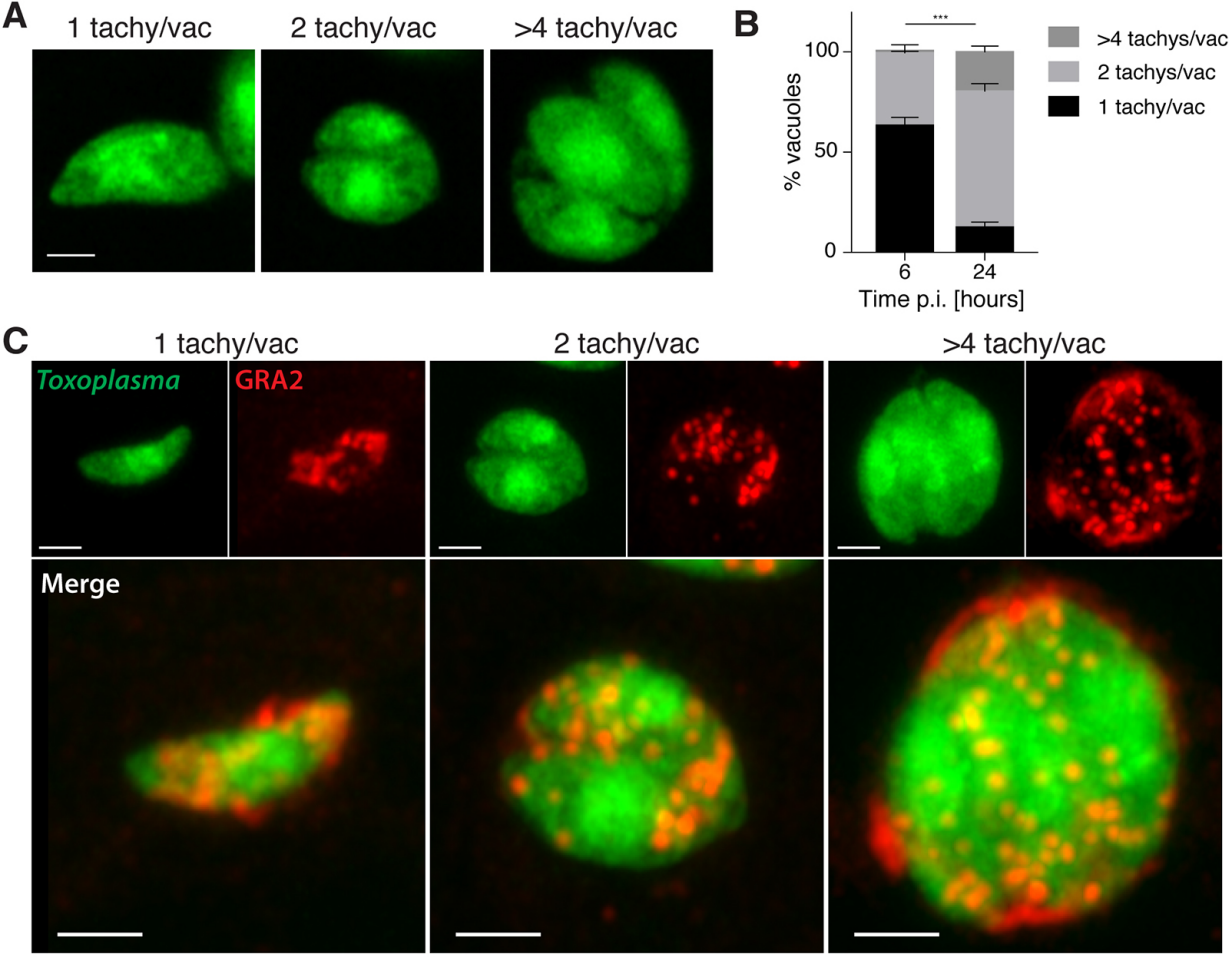
***Toxoplasma gondii* tachyzoites are intracellular and replicate in zebrafish.** (A) Representative images from AiryScan confocal imaging of replicating tachyzoites in fixed larvae infected in the HBV with type I *Toxoplasma-*GFP at 6 and 24 hpi, showing 1, 2 or >4 tachyzoites/vacuole. Scale bar: 2 µm. (B) Pixel volume quantification of individual GFP-positive punctae at 6 and 24 hpi. Significant differences (χ^2^_2_=58.5, ***P≤0.001) were observed between the percentage of total vacuoles counted in the HBV that are 1 tachyzoite/vacuole (<50 pix^3^), 2 tachyzoites/vacuole (50<100 pix^3^) or >4 tachyzoites/vacuole (>100 pix^3^) at 6 and 24 hpi. Pooled data from three independent experiments with at least three larvae per time point. Mean±s.e.m. shown. p.i., post-infection. (C) Representative AiryScan confocal images of replicating tachyzoites in larvae infected in the HBV with type I *Toxoplasma*-GFP (green; top-left images), fixed at 0, 6 and 24 hpi and labeled with α-GRA2 (red; top-right images) and merge (bottom large images). Shown are 1, 2 or >4 tachyzoites/vacuole. Scale bars: 2 µm.

To develop a *Toxoplasma*-zebrafish infection model, we tested whether tachyzoites could replicate in zebrafish larvae. We first used *Toxoplasma* type I (RH) strain, because it is known to grow faster *in vitro* and survive longer extracellularly than type II (Pru) and type III (CEP) strains (Saeij et al., 2005; Khan et al., 2009; Yang et al., 2013). We injected zebrafish larvae 3 days post-fertilization (dpf) in the hindbrain ventricle (HBV) with ∼5×10^3^ type I strain tachyzoites expressing GFP and followed infection for 24 h at 33°C (Fig. S1A). We observed parasite replication *in vivo* using time-lapse widefield fluorescent microscopy (Fig. S1B, Movie 1). Consistent with this, confocal microscopy showed that the percentage of vacuoles containing two or more tachyzoites significantly increased with time (Fig. 1A,B). To visualize PV formation around the replicating parasites, infected larvae were fixed and stained for granule antigen 2 (GRA2), a dense granule protein that accumulates in the PV lumen (Mercier et al., 2002). Here, GRA2 accumulated around single and replicating parasites, highlighting PV formation *in vivo* (Fig. 1C).

**Fig. 2.**
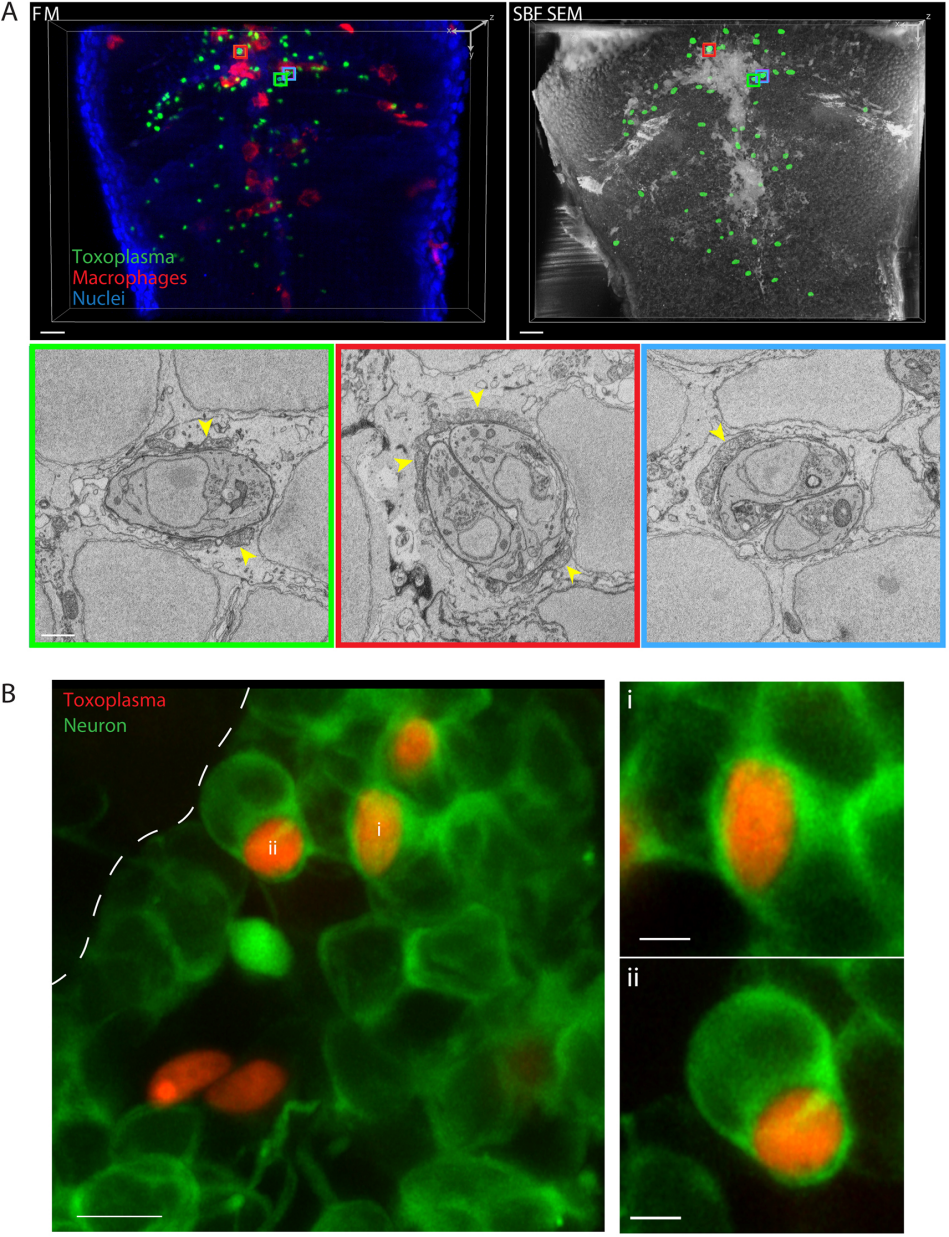
***Toxoplasma gondii* tachyzoites reside within zebrafish brain cells and neurons.** (A) 3D CLEM of tachyzoites in the HBV of transgenic *mpeg1*:*G/U*:mCherry larvae harboring macrophages (red) infected with type I *Toxoplasma-*GFP (green) at 6 hpi. 3D reconstructions of 40 confocal *z*-slices of a full vibratome section (FM, fluorescence microscopy; top left) and of 354 inverted consecutive 50 nm SBF SEM slices of a segment of it (top right). A middle slice of each of the *Toxoplasma* visible in the SBF SEM dataset was manually segmented (green; top right) to aid correlation. Regions of interest showing the localization of the high-resolution SBF SEM images (bottom row) are denoted with color boxes. Single (left; green box), replicating (middle; red box) and doublet (right; blue box) tachyzoites in zebrafish host cells were observed. See also Movie 2. Shown are three representative images out of 36 total *Toxoplasma* in zebrafish brain cells (see Fig. S2); tachyzoites were imaged in their whole volume to accurately determine their stage. Host mitochondrial recruitment to the parasitophorous vacuole is indicated by yellow arrowheads. Scale bars: 10 µm (top row) and 1 µm (bottom row). (B) Representative AiryScan confocal images of 3 dpf Tg(*elavl3*:*GCaMP6 s*)*^jf4^* larvae (neurons marked in green) infected in the HBV with type I *Toxoplasma-*Tomato (red) at 4 hpi. Shown are maximum projections of 35 *z*-slices (covering 5.98 µm) out of 85 slices imaged (left, five tachyzoites total imaged). The ventricular surface is highlighted by a white dashed line. Of the three tachyzoites found within green neurons from the left image, shown are magnified maximum-projection images of two tachyzoites covering 8 (i, top right) or 17 (ii, bottom right) *z*-slices (*z*=0.17 µm) out of 85 total. Scale bars: 5 µm (left image) and 2 µm (right images). Two of the five tachyzoites imaged are not inside green neurons (left) and close to the ventricular surface where green neurons become sporadic (see Movie 3), which suggests active invasion of progenitors/ependymal cells.

To investigate parasite morphology and location at 6 h post-infection (hpi), 3D CLEM using serial blockface scanning electron microscopy (SBF SEM) was performed on the HBV of infected zebrafish. We observed 39 parasites (from *n*=2 larvae) located inside cells of the zebrafish hindbrain, which all displayed host mitochondrial association (Fig. 2A; Fig. S2, Movie 2; see also Fig. 6), a hallmark of intracellular type I parasites previously described in mouse and human cells (Pernas et al., 2014). Moreover, tachyzoites could be observed as singlets, replicating doublets (thus joined together) or fully replicated doublets (two tachyzoites not joined together) (Fig. 2A; Fig. S2, Movie 2).

To characterize the specific cell type within which type I parasites reside and replicate in the zebrafish hindbrain, we used Tg(*elavl3*:*GCaMP6s*)*^jf4^* transgenic larvae. The *elavl3* promoter drives expression in post-mitotic CNS cells fated to become neurons (Park et al., 2000). Embryos harboring this transgene were infected with type I-Tomato parasites (Fig. 2B; Movie 3). In agreement with 3D CLEM data showing that tachyzoites reside within brain cells, infection of *elavl3*:*GCaMP6s* transgenic larvae revealed that ∼1/3 of tachyzoites (5/14 and 8/25 from *n*=2 larvae) reside within GFP^+^ cells at 4 hpi. Collectively, these results show that type I *Toxoplasma* tachyzoites can invade zebrafish cells and replicate *in vivo*, and that tachyzoites favor brain cells over myeloid cells in the zebrafish hindbrain.

### Type II and III parasites are more efficient than type I parasites at establishing infection *in vivo*

**Fig. 3.**
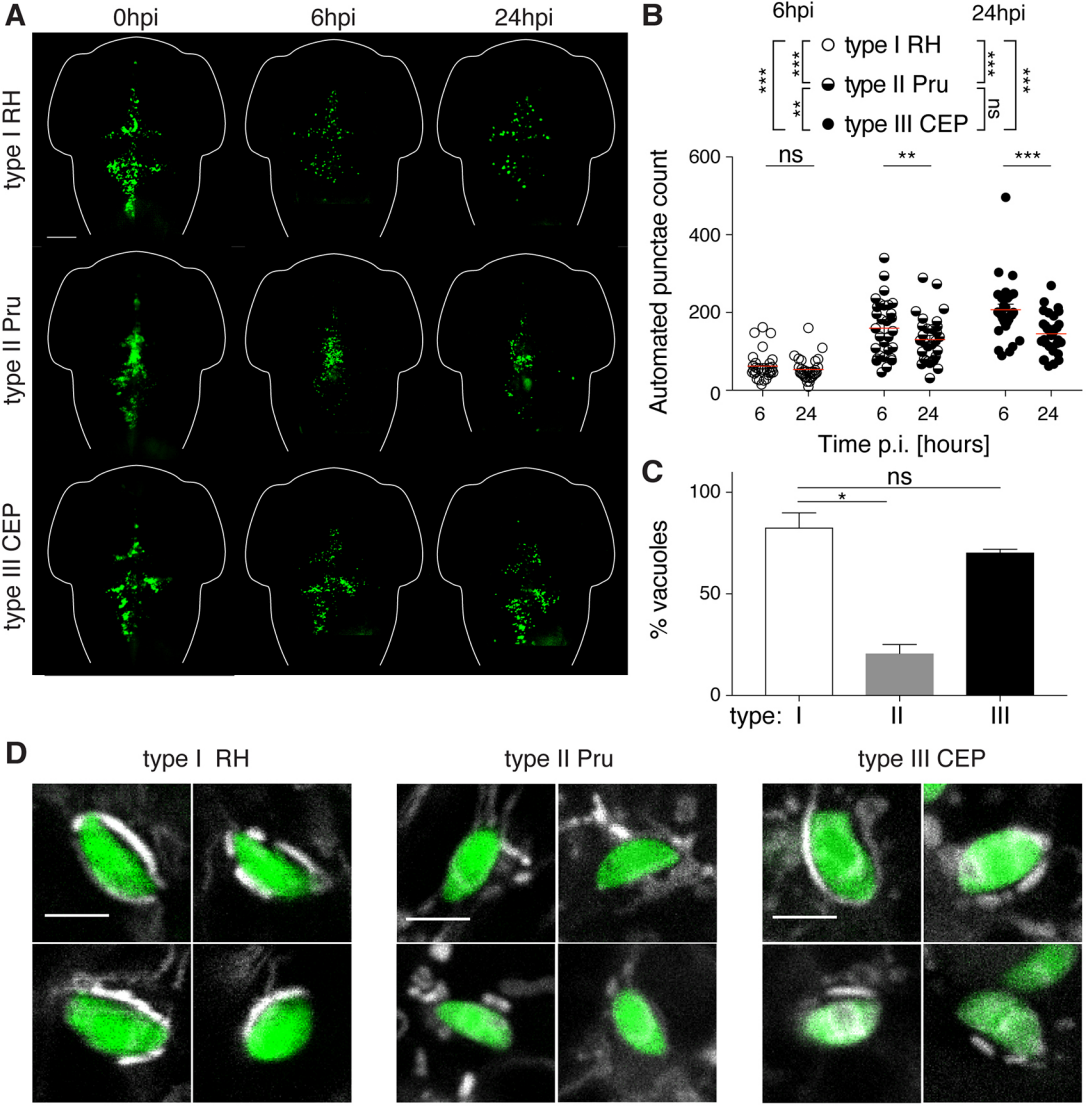
**Non-lethal zebrafish larvae model of acute *Toxoplasma gondii* infection.** (A) Representative images of larvae infected in the HBV with type I (RH; top row), type II (Pru; middle row) or type III (CEP; bottom row) of *Toxoplasma* (green). Individual larvae were imaged and monitored at 0, 6 and 24 hpi by fluorescent stereomicroscopy. Scale bar: 100 µm. (B) Automated enumeration of GFP-positive punctae at 6 hpi and 24 hpi of larvae infected with type I (RH; open circles), type II (Pru; semi-closed circles) or type III (CEP; closed circles) of *Toxoplasma* tachyzoites. Automated counts were supported by manual quantifications (Fig. S3C). Mean±s.e.m. shown. Pooled data from at least three independent experiments with at least five larvae per condition per experiment. Significance calculated using two-way ANOVA (repeated measures) with Sidak's multiple comparisons test. ns, *P*>0.05; **P≤0.01, ***P≤0.001. p.i., post-infection. (C) Quantification of the percentage of type I (white bar), type II (gray bar) or type III (black bar) vacuoles exhibiting host mitochondrial association at 6 hpi in the zebrafish hindbrain. Significant differences were observed between the parasite strains (Kruskal–Wallis *P*=0.0036), with type II parasites shown to be lower (20±4.3%) than type I (82±7.2%) or type III (70±1.6%) parasites. Significance calculated using Dunn's multiple comparisons test. ns, *P*>0.05; *P≤0.05. Pooled data from at least three independent experiments with three larvae per condition per experiment. Mean±s.e.m. shown. (D) Representative confocal images of larvae infected in the HBV with type I (RH; left panels), type II (Pru; middle panels) or type III (CEP; right panels) of *Toxoplasma* (green) and stained for mitochondria (white) at 6 hpi, showing four examples each of host mitochondrial recruitment (for type I and type III) or no host mitochondrial recruitment (for type II). Scale bars: 5 µm.

To determine if parasite strain can affect parasite burden and host response in our zebrafish model, we infected larvae with ∼5×10^3^ type I, II or III *Toxoplasma*-GFP (Fig. S3A). In all cases, infected larvae showed 100% survival and no adverse effects up to 48 hpi (Fig. S3B). To quantify parasite burden in a high-throughput manner, we optimized an automated quantification pipeline using ZedMate (Yakimovich et al., 2019 preprint) for the different strain types at 6 hpi and 24 hpi. Strikingly, type II and III parasite burden was ∼3× higher than type I parasite burden at 6 hpi (Fig. 3A,B; Fig. S3C). Analysis by fluorescent stereomicroscopy showed that, from the initial parasite input of ∼5×10^3^ tachyzoites (Fig. S3A), parasite burden was reduced ∼95% by 6 hpi, suggesting that ∼5% of parasites (∼250 tachyzoites, Fig. 3B; Fig. S3C) successfully invade zebrafish cells and establish infection. Once established at 6 hpi, all three strain types persisted equally and decreased by ∼20% between 6 hpi and 24 hpi.

To test if host mitochondrial association was observed across the three strain types, we stained host mitochondria in the HBV of infected zebrafish larvae. In agreement with *in vitro* observations (Pernas et al., 2014), ∼82% and ∼70% of type I and type III parasites, respectively, showed clear host mitochondrial association, while only ∼20% of type II parasites showed clear host mitochondria association (Fig. 3C,D). These results demonstrate that strain type-dependent host mitochondrial association characteristics are conserved in zebrafish *in vivo*.

### Macrophage and neutrophil response to parasite infection *in vivo*

**Fig. 4.**
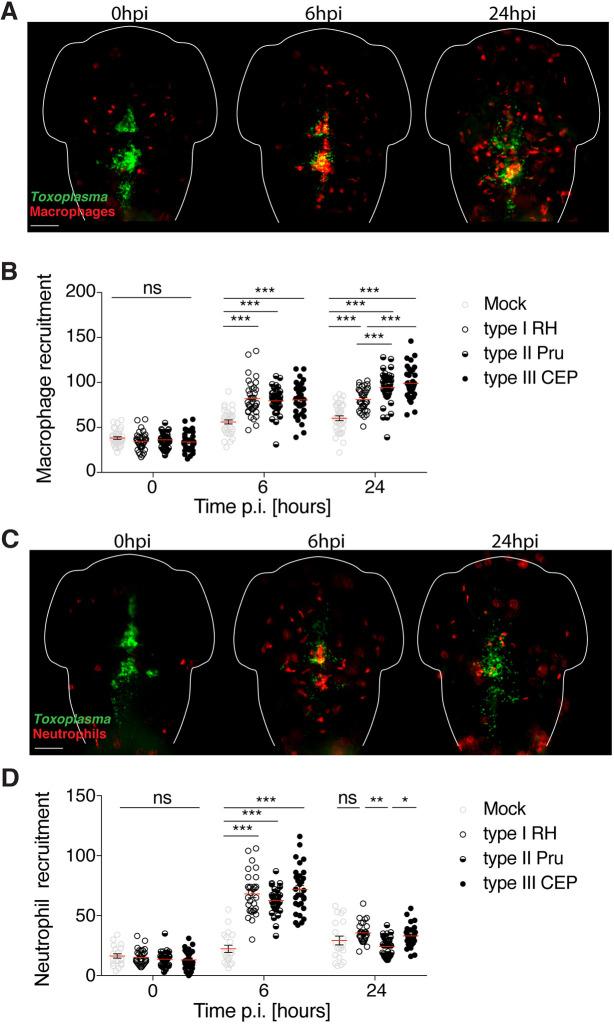
**Leukocyte recruitment to *Toxoplasma gondii in vivo*.** (A) Representative images of *mpeg1*:*G/U*:mCherry larvae harboring macrophages (red) infected in the HBV with *Toxoplasma* (green). Individual larvae were imaged and monitored at 0, 6 and 24 hpi by fluorescent stereomicroscopy. Scale bar: 100 µm. (B) Quantification of macrophages in *mpeg1*:*G/U*:mCherry larvae at 0, 6 and 24 hpi injected with mock [human foreskin fibroblast (HFF) lysate; gray open circles], type I (RH; open circles), type II (Pru; semi-closed circles) or type III (CEP; closed circles) parasites. Pooled data from at least three independent experiments with at least seven larvae per condition per experiment. Mean±s.e.m. shown. Significance calculated using two-way ANOVA (repeated measures) with Sidak's multiple comparisons test. ns, *P*>0.05; ***P≤0.001. p.i., post-infection. (C) Representative images of *lyz*:dsRed larvae harboring neutrophils (red) infected in the HBV with *Toxoplasma* (green). Individual larvae were imaged and monitored at 0, 6 and 24 hpi by fluorescent stereomicroscopy. Scale bar: 100 µm. (D) Quantification of neutrophils in *lyz*:dsRed larvae at 0, 6 and 24 hpi injected with mock (HFF lysate; gray open circles), type I (RH; open circles), type II (Pru; semi-closed circles) or type III (CEP; closed circles). Pooled data from at least two independent experiments with at least three larvae per condition per experiment. Mean±s.e.m. shown. Significance calculated using two-way ANOVA (repeated measures) with Tukey's multiple comparisons test. ns, *P*>0.05; *P≤0.01, **P≤0.01, ***P≤0.001.

To analyze *Toxoplasma*-macrophage interactions over time, 3 dpf transgenic larvae possessing red macrophages Tg(*mpeg1*:*Gal4-FF*)*^gl25^/*Tg(*UAS-E1b*:*nfsB*.mCherry)*^c264^* (herein referred to as *mpeg1*:*G/U*:mCherry), were infected with type I, II or III *Toxoplasma*-GFP, and macrophage recruitment was quantified by fluorescent stereomicroscopy. Compared to mock injection, the number of macrophages recruited to the infection site was significantly increased (∼1.5 fold) for all three strain types at both 6 hpi and 24 hpi (Fig. 4A,B).

To analyze *Toxoplasma*-neutrophil interactions over time, 3 dpf transgenic larvae possessing red neutrophils Tg(*lyz*:dsRed)^*nz50*^ (herein referred to as *lyz*:dsRed), were infected with type I, II or III *Toxoplasma*-GFP, and neutrophil recruitment was quantified by fluorescent stereomicroscopy. Here, the number of neutrophils recruited to the infection site was significantly increased (∼3 fold) compared to mock injection for all three strain types at 6 hpi (Fig. 4C,D). In contrast to macrophages, which remained at the infection site by 24 hpi, the number of neutrophils recruited to the infection site (for all three strain types) decreased significantly, reaching basal levels by 24 hpi.

### Macrophages control parasite burden *in vivo*

**Fig. 5.**
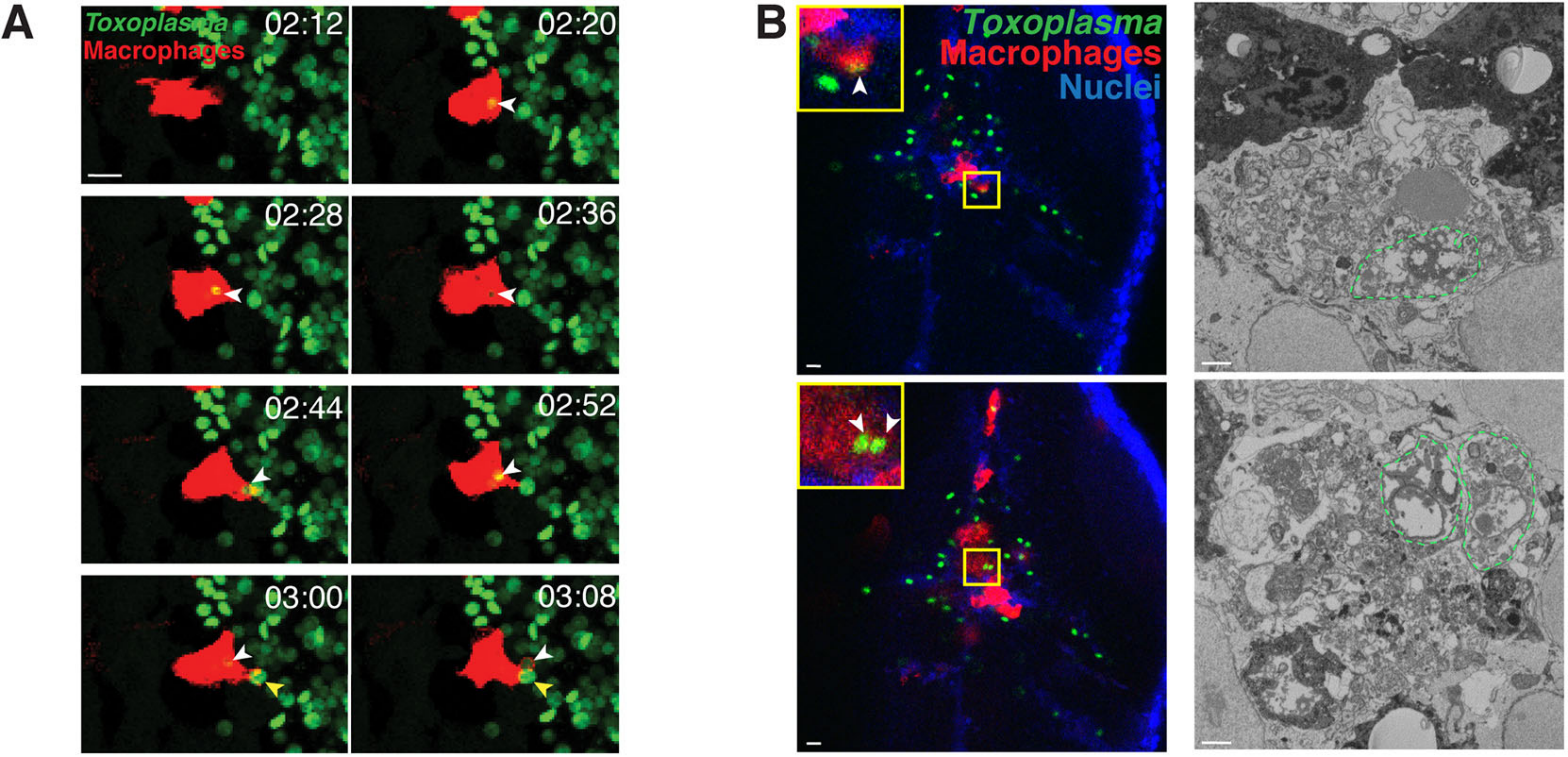
**Macrophages phagocytose and degrade *Toxoplasma gondii in vivo*.** (A) Representative frames extracted from *in vivo* confocal imaging of *mpeg1*:*G/U*:mCherry larvae harboring macrophages (red) injected with type I *Toxoplasma-*GFP (green). First frame at 2 h 12 min post-infection (mpi) followed by seven consecutive frames taken at 8 min intervals. Shown are maximum projections of 24 *z*-slices taken at 2 µm optical sections. White arrowheads label a phagocytosed parasite at 2 h 20 mpi that loses its green fluorescence by 3 h 8 mpi. Yellow arrowheads indicate a new phagocytosis event of a green parasite at 3 hpi to 3 h 8 mpi. Scale bar: 10 µm. See also Movie 5. (B) 3D CLEM of dead/dying tachyzoites in the HBV of *mpeg1*:*G/U*:mCherry larvae harboring macrophages (red) infected with type I *Toxoplasma-*GFP (green) at 6 hpi and stained with Hoechst 33342 (blue). Representative images extracted from confocal *z*-stacks of a full vibratome section (left column) and from consecutive 50 nm SBF SEM slices of a segment of it (right column). Dead/dying parasites are indicated by white arrowheads (insets, left column) and outlined by green dashed lines (right column). Scale bars: 10 µm (left column) and 1 µm (right column).

To analyze the interactions between type I *Toxoplasma* and macrophages in depth, we imaged infected *mpeg1*:*G/U*:mCherry larvae with *Toxoplasma-*GFP at 6 hpi by 3D CLEM. Of the 18 tachyzoites found inside macrophages (from *n*=3 larvae), seven were intact parasites that had actively invaded macrophages, as identified by host mitochondrial association to the membrane surrounding the parasites (Fig. S4). Six of the seven intact tachyzoites were single tachyzoites inside PVs. This suggests that macrophages may prevent parasite replication. Conversely, a single event captured by 3D CLEM showed *Toxoplasma* replication within a zebrafish macrophage, as judged by host mitochondrial association to the vacuolar membrane and the presence of two replicating tachyzoites (still joined together), with one of the replicating tachyzoites harboring two nuclei (Fig. S5A,B, Movie 4). To follow the fate of type I parasites engulfed by macrophages in real time, *mpeg1*:*G/U*:mCherry larvae infected with *Toxoplasma-*GFP were imaged by time-lapse confocal microscopy. Here, we captured the movement of macrophages in the hindbrain harboring type I parasites that had been actively invaded, as identified by host mitochondrial association (Fig. S5C), suggesting that, in some cases, macrophages may facilitate parasite movement in the brain tissue. However, we also observed parasite engulfment by macrophages followed by loss of GFP fluorescence, suggesting active parasite degradation (Fig. 5A; Movie 5). Consistent with this, 3D CLEM showed parasite degradation inside macrophages, as identified by fragmentation of tachyzoite organelles (Fig. 5B; Fig. S6).

**Fig. 6.**
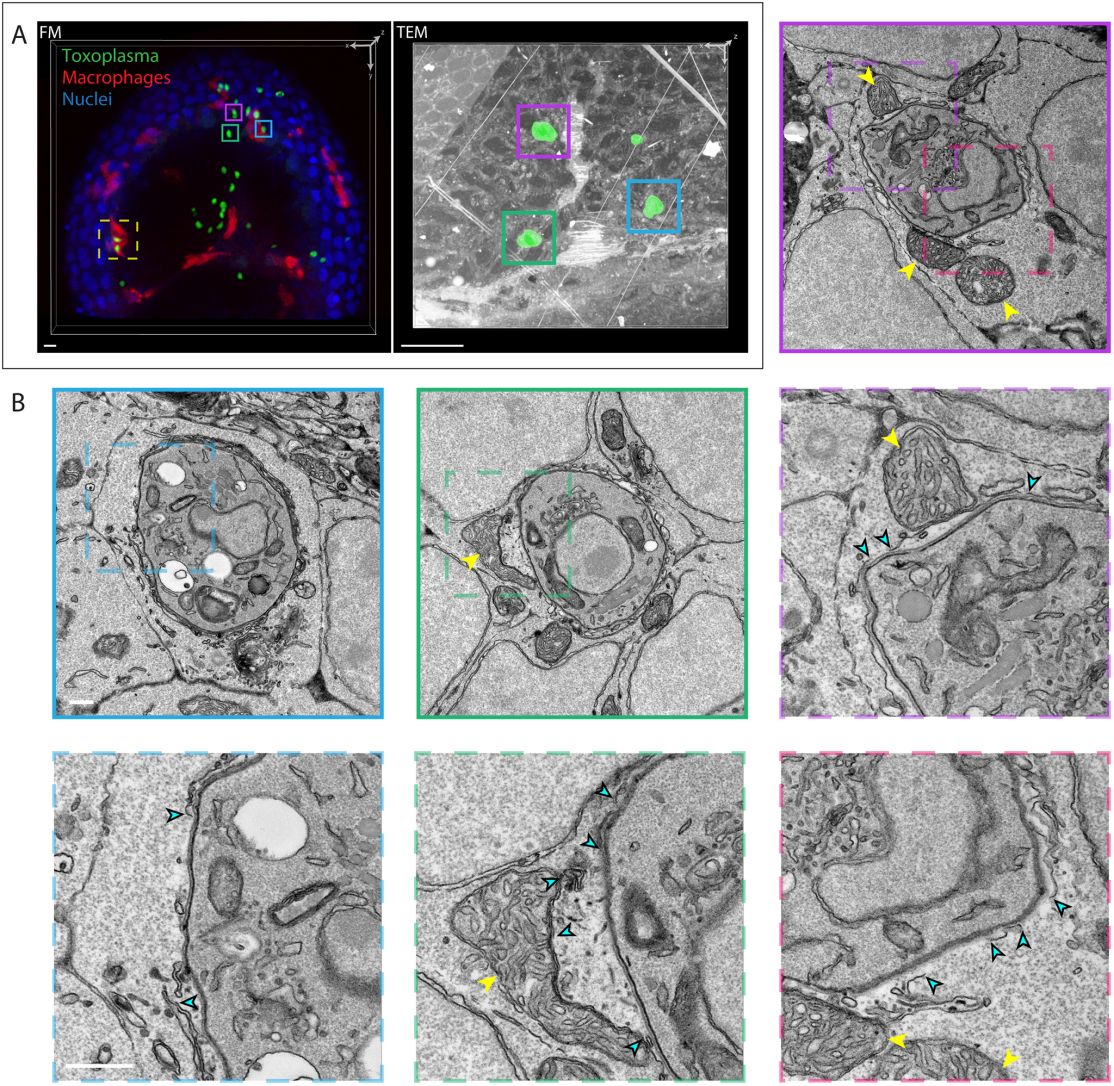
**Host cell-intrinsic response in zebrafish disrupts *Toxoplasma gondii* parasitophorous vacuoles in brain cells *in vivo.*** (A) 3D CLEM of tachyzoites in the HBV of transgenic *mpeg1*:*G/U*:mCherry larvae harboring macrophages (red) infected with type I *Toxoplasma-*GFP (green) at 6 hpi and stained with Hoechst 33342 (blue). 3D reconstructions of 59 confocal *z*-slices (40×) of a part of the HBV (FM, fluorescence microscopy; left) and of 75 inverted consecutive 70 nm sections imaged by ssTEM at 440× magnification (right). Each of the *Toxoplasma* visible in the ssTEM dataset was manually segmented (green; right) in every section to aid correlation. Regions of interest showing the localization of the high-resolution ssTEM images shown in B (cyan, magenta and green boxes) and Fig. 7 (yellow dashed line box) are indicated. Scale bars: 10 µm. (B) Representative higher-magnification ssTEM images of *Toxoplasma* in HBV cells. Continuous line boxes show 6800× magnification images; dashed line boxes show 18,500× magnification images. *Toxoplasma* tachyzoites were imaged in their full volume to accurately assess the continuity of the PV. See also Movie 6. Host mitochondrial recruitment to the PV is indicated by yellow arrowheads; breaks in the PV are indicated by cyan arrowheads. Scale bars: 1 µm (continuous line box) and 500 nm (dashed line box).

**Fig. 7.**
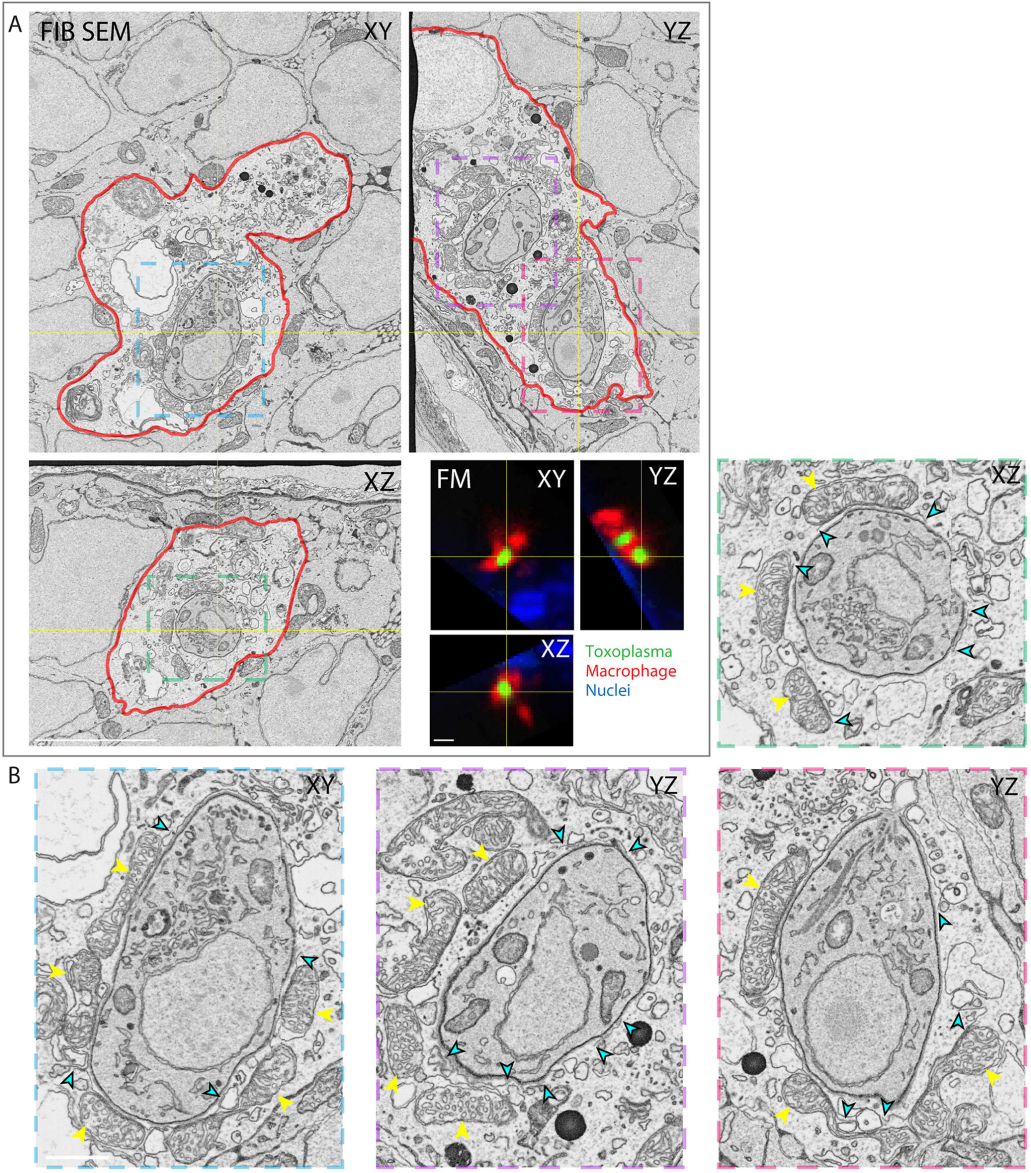
**Host cell-intrinsic response disrupts *Toxoplasma gondii* parasitophorous vacuoles in zebrafish macrophages *in vivo.*** (A) 3D CLEM of two parasites inside a macrophage in the HBV of *mpeg1*:*G/U*:mCherry larvae harboring macrophages (red) infected with type I *Toxoplasma*-GFP (green) at 6 hpi and stained with Hoechst 33342 (blue). Orthoslices of 3200 consecutive 5 nm FIB SEM slices of the whole macrophage (top left; see also Movie 7) and of 59 confocal *z*-slices (40×) re-sliced to match the orientation of the FIB SEM data (FM, bottom right; cell also shown in yellow dashed line box in Fig. 6A). Color boxes show localization of the cropped and enlarged images of tachyzoites shown in B. The plasma membrane of the macrophage (red) was manually segmented to aid correlation. (B) Cropped and enlarged images of the *Toxoplasma* shown in the FIB SEM orthoslices in A. Host mitochondrial recruitment to the PV is indicated by yellow arrowheads; breaks in the PV are indicated by cyan arrowheads. Scale bars: 5 µm (A) and 1 µm (B).

To investigate the structure of the PV surrounding tachyzoites inside brain cells and macrophages in the hindbrain, we imaged *mpeg1*:*G/U*:mCherry larvae infected with *Toxoplasma-*GFP using high-resolution correlative serial section transmission electron microscopy (ssTEM) and focused ion beam scanning electron microscopy (FIB SEM). At 6 hpi, ssTEM revealed gaps in the PV around type I tachyzoites in brain cells (Fig. 6; Movie 6). Consistent with this, and reports of vacuole breakage in human macrophages (Fisch et al., 2020 preprint), FIB SEM also captured incomplete vacuole membranes around type I tachyzoites within a macrophage (Fig. 7; Movie 7). Together, ssTEM and FIB SEM demonstrate that cell-intrinsic host immune pathways may be activated within both zebrafish brain cells and macrophages in order to control *Toxoplasma* infection.

**Fig. 8.**
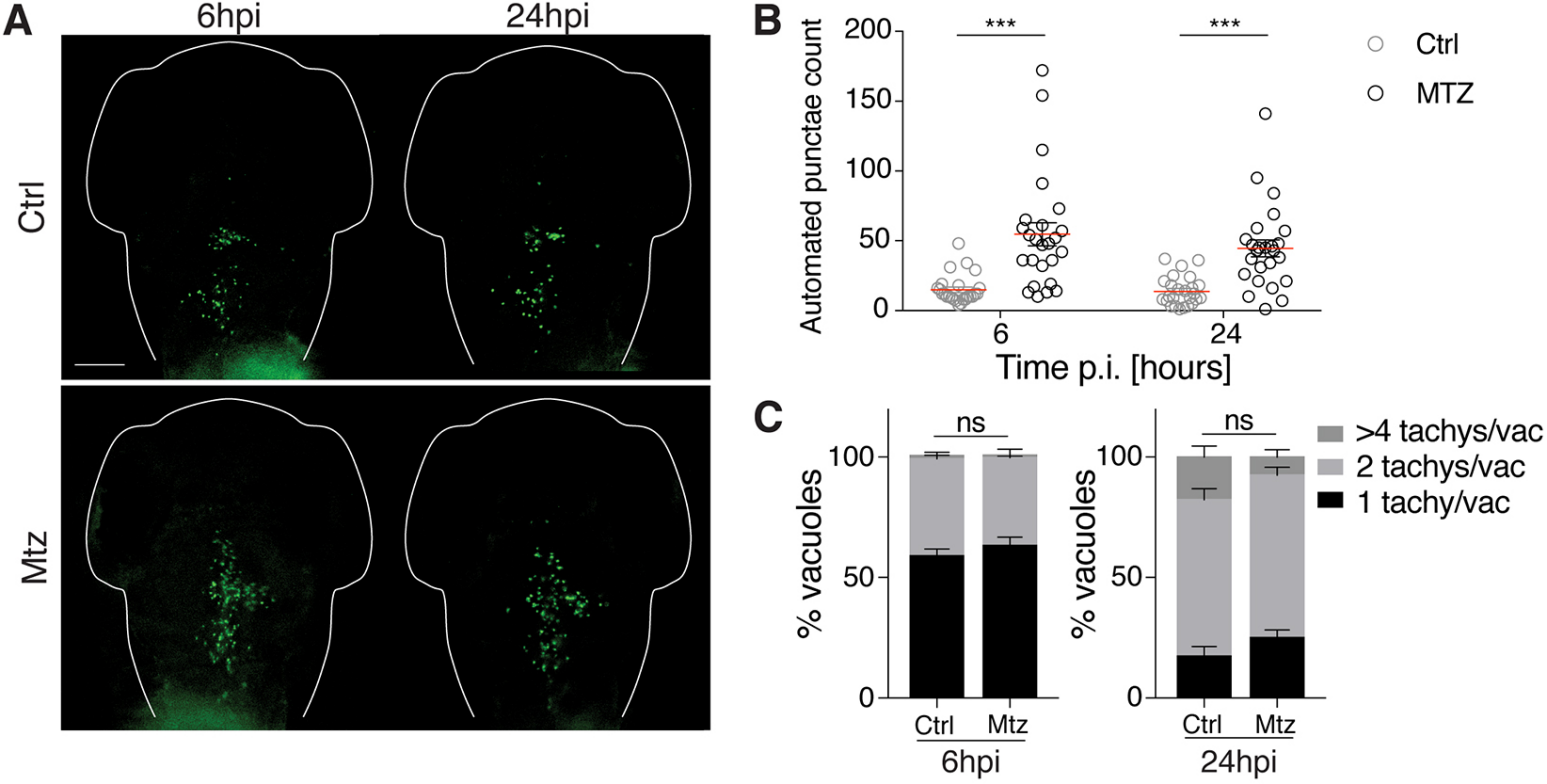
**Macrophages control *Toxoplasma gondii* burden *in vivo*.** (A) Representative images of control (Ctrl; top) or macrophage-ablated (Mtz; bottom) *mpeg*:*G/U*:mCherry larvae infected in HBV with type I *Toxoplasma-*GFP (green). Individual larvae were imaged at 6 hpi and 24 hpi by fluorescent stereomicroscopy. Scale bar: 100 µm. (B) Automated enumeration of GFP-positive punctae in the HBV at 6 hpi and 24 hpi of Ctrl (gray open circles) or macrophage-ablated (open circles) larvae infected with type I *Toxoplasma* tachyzoites. Pooled data from three independent experiments with at least seven larvae per condition per experiment. Significance calculated using two-way ANOVA (repeated measures) with Sidak's multiple comparisons test. ***P≤0.001. p.i., post-infection. (C) Pixel volume quantification of individual GFP-positive punctae in Ctrl or macrophage-ablated larvae at 6 hpi and 24 hpi. Presented as percentage of total vacuoles counted in the HBV that are 1 tachyzoite/vacuole (<50 pix^3^), 2 tachyzoites/vacuole (50<100 pix^3^) or >4 tachyzoites/vacuole (>100 pix^3^). Pooled data from three independent experiments with at least three larvae per time point. Significance calculated using Chi-square test, χ^2^_2_=1.248 (6 hpi), χ^2^_2_=5.4 (24 hpi). ns, *P*>0.05. Mean±s.e.m. shown.

To test the role of macrophages in *Toxoplasma* infection at the whole-organism level, we used the transgenic line Tg(*mpeg1*:*Gal4-FF*)*^gl25^/*Tg(*UAS-E1b*:*nfsB*.mCherry)*^c264^*, which enables the specific ablation of macrophages upon metronidazole treatment (Fig. S7A,B). In the absence of macrophages, infected larvae showed 100% survival (Fig. S7C). However, upon infection of ∼5×10^3^ type I tachyzoites, parasite burden was significantly increased in the absence of macrophages at both 6 hpi and 24 hpi, suggesting that macrophages are responsible for parasite clearance *in vivo* (Fig. 8A,B; Fig. S7D,E). Similarly, a significant increase in parasite burden in macrophage-ablated larvae (compared to control larvae) was observed upon infection of ∼5×10^3^ type II and III tachyzoites (Fig. S7F,G). To analyze the viability of parasites that are cleared by macrophages, we performed pixel volume quantification, and show that control and metronidazole-treated larvae contain equally replicating *Toxoplasma* tachyzoites (% of total vacuoles counted in the HBV; Fig. 8C). These data suggest that macrophages have a dominant role in clearing healthy, viable parasites rather than supporting their replicative niche.

## DISCUSSION

Zebrafish infection models for studying eukaryotic parasites are beginning to emerge (Gomes and Mostowy, 2020; Dóró et al., 2019). In this study, we established a novel *Toxoplasma* infection model using zebrafish larvae to explore host-parasite interaction *in vivo* in the hindbrain. We found that the three main clonal lineages of *Toxoplasma* are able to establish infection in the zebrafish hindbrain and replicate within their PV, and reveal that macrophages are key in controlling viable parasites *in vivo*. Using the type I strain we showed that *Toxoplasma* invade zebrafish brain cells (including post-mitotic cells fated to become neurons, expressing GCaMP6s driven by the *elavl3* promoter). Additionally, using state-of-the-art electron microscopy techniques, we observed vacuole breakage around tachyzoites in both zebrafish brain cells and macrophages *in vivo*.

Using time-lapse confocal microscopy and 3D CLEM, we visualized single and replicating type I tachyzoites in the zebrafish HBV exhibiting host mitochondrial association. The relatively slow replication cycle of *Toxoplasma* observed in tissue culture cells *in vitro* (>6 h) is consistent with that observed in the zebrafish HBV. GRA2 staining and 3D CLEM of replicating tachyzoites strongly suggests the formation of a PV. This is indicative of normal type I parasite behavior, as demonstrated *in vitro* using tissue culture cells and *in vivo* using other animal models (Black and Boothroyd, 2000).

The zebrafish HBV is well established to investigate host response to infection (Yoshida et al., 2017; Torraca and Mostowy, 2018; Gomes and Mostowy, 2020). We did not observe *Toxoplasma* dissemination from the HBV, and this allowed us to monitor leukocyte-parasite interactions within a localized area. Here, type II and III strains were more efficient than type I strains at maintaining a higher infectious burden. This suggests that type II and III strains may be more efficient at invading non-phagocytic cell types found in the HBV and/or evading clearance by host cells. Together with 3D CLEM and confocal microscopy, we conclude that *Toxoplasma* can invade non-phagocytic cells (with ∼1/3 of parasites within post-mitotic neurons) in the HBV of zebrafish larvae. Although mortality was not observed in our 24 h *Toxoplasma* infection model, it will be of great interest to explore the long-term consequences of *Toxoplasma* infection on parasite dissemination and zebrafish survival.

Both live-cell imaging and 3D CLEM showed type I parasite uptake and clearance by macrophages. In all cases of macrophage-parasite interaction captured by 3D CLEM, macrophages retained their fluorescence during *Toxoplasma* infection and had intact nuclei and mitochondria, indicative of a healthy host cell. Conversely, phagocytosed tachyzoites (i.e. those that had not entered by active invasion) exhibited no host mitochondrial association, which suggests no PV formation, and showed loss of organelle integrity. Our evidence obtained from time-lapse microscopy, 3D CLEM and macrophage ablation experiments highlight that active parasite clearance by zebrafish macrophages *in vivo* occurs within the first 6 hpi. Using ssTEM and FIB SEM, we observed incomplete PV membrane in zebrafish brain cells and macrophages. PV breakage has been observed *in vitro* after IFN-γ stimulation of murine cells and human macrophages, and is thought to be part of the cell-intrinsic host defense mechanism against *Toxoplasma* tachyzoites (Martens et al., 2005; Zhao et al., 2008; Yamamoto et al., 2012; Selleck et al., 2015; Fisch et al., 2020 preprint). Zebrafish possess an ortholog of IFN-γ together with a related gene (IFN-γ-rel), and studies have suggested that zebrafish IFN-γ (also known as Ifng1) expression is associated with host protection against bacterial infection, while IFN-γ-rel expression is associated with host protection against viral challenge (Igawa et al., 2006; López-Muñoz et al., 2009, 2011; Sieger et al., 2009; Yoon et al., 2016). Therefore, future work using our zebrafish infection model could explore the precise anti-parasitic mechanisms (including the role of IFN-γ and IFN-γ-rel) employed by brain cells and macrophages during *Toxoplasma* infection.

In murine *in vivo* models, various leukocytes have been implicated in trafficking *Toxoplasma* from the site of infection. Examples include infected neutrophils that pass from the intestine to the lumen (Coombes et al., 2013), as well as infected macrophages and dendritic cells that pass the blood brain barrier (Courret et al., 2006). It is intriguing, therefore, to note that our study using time-lapse microscopy showed parasite-infected macrophages moving through brain tissue for possible parasite transport (Fig. S5C). Analysis of our 3D CLEM data also identified an actively replicating type I parasite inside a macrophage exhibiting association of host mitochondria with the PV (Fig. S5A,B). This observation is reminiscent of parasite replication ‘hot spots’ described *in vivo* in the murine intestinal villi (Coombes et al., 2013). Injection of tachyzoites directly into the hindbrain of zebrafish is likely to promote *Toxoplasma* infection of neurons [its favored long-term niche (Ferguson and Hutchison, 1987; Cabral et al., 2016)], and may explain the lack of parasite dissemination by macrophages observed in our model. Overall, it is remarkable that zebrafish macrophages during *Toxoplasma* infection *in vivo* have the capacity to phenocopy known behavior exhibited by murine macrophages during *Toxoplasma* infection *in vivo*. This is the case for both the type of event observed (e.g. parasite killing, trafficking, sustaining replication) and their approximate *in vivo* frequency.

In summary, we have established a novel animal model for studying the *in vivo* innate immune response to *Toxoplasma* infection, and for comparing host response to the three main *Toxoplasma* strain types *in vivo*. We also demonstrate a dominant role for macrophages in parasite clearance. Having established a zebrafish model of *Toxoplasma* infection, we have revealed a unique *in vivo* infection platform for CRISPR targeting and high-throughput drug screens that, together with time-lapse microscopy, can be used to identify determinants underlying *Toxoplasma* infection control.

## MATERIALS AND METHODS

### Ethics statement

Animal experiments were performed according to the Animals (Scientific Procedures) Act 1986 and approved by the Home Office (project licenses PPL P84A89400 and P4E664E3C). All experiments were conducted up to 5 dpf.

### Zebrafish husbandry and maintenance

Fish were reared and maintained at 28.5°C on a 14 h light, 10 h dark cycle. Embryos obtained by natural spawning were maintained in 0.5× E2 medium supplemented with 0.3 µg/ml Methylene Blue. Larvae were anesthetized with 20 µg/ml tricaine (Sigma-Aldrich) during the injection procedures and for live *in vivo* imaging. All experiments were carried out on TraNac background (Krauss et al., 2013) larvae to minimize obstruction of fluorescence signal by pigmentation leading to misrepresentation in parasite dose quantification.

### Parasite culture, preparation and infection

*Toxoplasma* (RH/Pru/CEP) expressing GFP/luciferase or Tomato was maintained *in vitro* by serial passage on human foreskin fibroblast (HFF) cultures (American Type Culture Collection, CVCL_3285). Cultures were grown in Dulbecco's modified Eagle medium high glucose (Life Technologies) supplemented with 10% fetal bovine serum (FBS; Life Technologies) at 37°C in 5% CO_2_. Parasites were prepared from 25G followed by 27G syringe-lysed HFF cultures in 10% FBS. Excess HFF material was removed by centrifugation for 10 min at 50 ***g***. After washing with PBS, *Toxoplasma* tachyzoites were resuspended in PBS at 2×10^6^ tachyzoites/µl. During injection, tachyzoites were maintained at room temperature and passed through a 29G myjector syringe (Terumo) to dissociate clumps and homogenize the suspension. Control infections were carried out using uninfected HFF cultures prepared as described above. Larvae at 3 dpf were anesthetized and injected with ∼2 nl parasite suspension into the HBV. HBV injections were carried out as previously described (Mazon Moya et al., 2017). Larvae are optimally maintained at 28.5°C, but develop normally between 23°C and 33°C (Westerfield, 2007). *Toxoplasma* invades and replicates at a minimum of 33°C. Infected larvae were therefore transferred into medium pre-warmed to 33°C to ensure normal zebrafish development and parasite replication (Fig. S1A). Progress of infection was monitored by fluorescent stereomicroscopy (Leica M205FA, Leica Microsystems).

### Quantification of parasite dose and burden

For parasite dose quantification, *z*-stacks of the infected hindbrain were taken within 5-10 min using the Leica M205FA fluorescent stereomicroscope at 130× magnification using a 1× objective. Images were analyzed using the particle analysis function in Fiji software (Schindelin et al., 2012). For manual quantification of parasite burden, *z*-stacks were taken using the Leica M205FA. GFP-positive punctae were quantified using the multi-point tool in Fiji software. Computer vision-driven automated parasite burden quantifications were carried out using the ZedMate plugin in Fiji software to corroborate manual quantifications (Yakimovich et al., 2019 preprint). Pixel volume quantifications were carried out by 3D projecting confocal *z*-stacks and using the 3D objects counter tool in Fiji (Movie 8). For Movie 8, scale was increased from 124×165 pixels (11 pixels=4.8 µm) to 1240×1650 pixels for clarity.

### Live imaging, image processing and analysis

Live *in vivo* imaging was performed on anesthetized larvae immobilized in 1% low-melting-point agarose in 35 mm glass-bottomed dishes (MatTek). Widefield microscopy was performed using a 40× objective. *Z*-stacks were acquired at 10 min intervals. Sixty *z*-slices were taken at 2 µm sections. Post-acquisition, scale was increased from 155×155 pixels (100.75×100.75 µm) to 1000×1000 pixels and further cropped to a final size of 204×204 pixels around the region of interest (ROI). Confocal microscopy was performed using the Zeiss Invert LSM 710 (Carl Zeiss AG) and the LSM 880 (Carl Zeiss AG) using a 40× and 63× objective. *Z*-stacks were acquired at 8 min intervals. Sixty *z*-slices were taken at 0.9 µm sections per larva. For all time-lapse acquisitions, larvae were maintained at 33°C. For mitochondria staining, larvae were injected with 1 nl MitoTracker^®^ DeepRed (250 µM; Life Technologies) 40 min prior to embedding for live confocal microscopy. For characterization of the cell type that *Toxoplasma* tachyzoites are invading, the Tg(*elavl3*:*GCaMP6s*)*^jf4^* transgenic line was used. This particular transgenic line employs an enhanced GFP protein variant fused to calcium-binding proteins to read out neuronal calcium flux (Vladimirov et al., 2014); however, we did not employ the calcium sensitivity functionality for our experiments: it was used purely to identify neurons. For confocal microscopy of Tg(*elavl3*:*GCaMP6s*)*^jf4^* larvae, LSM 880 (Carl Zeiss AG) was used with the 63× objective. For Movie 3, a confocal *z*-stack of 33 *z*-slices (*z*=0.43 µm) was acquired. Post-acquisition, scale was increased from 1232×1232 pixels (104.84×104.84 µm) to 2000×2000 pixels. For Fig. 2B, a *z*-stack of 85 slices (covering 14.525 µm) was acquired using the AiryScan super-resolution (SR) mode and processed using the AiryScan processing tool in Zen Blue (Carl Zeiss AG).

### Wholemount immunohistochemistry

Euthanized larvae were fixed overnight at 4°C in 4% paraformaldehyde supplemented with 0.4% Triton X-100 and washed in PBS, 0.4% Triton X-100 before staining. Briefly, after a 20 min wash in PBS 1% Triton X-100, larvae were incubated overnight at 4°C in blocking solution: PBS supplemented with 10% FBS, 1% dimethyl sulfoxide (DMSO) and 0.1% Tween 20. Primary antibody diluted 1:1000 in blocking solution was applied overnight at 4°C. Larvae were washed 4×15 min with PBS supplemented with 0.1% Tween 20. Secondary antibody diluted 1:500 in blocking solution was applied overnight at 4°C. Larvae were washed 4×15 min with PBS supplemented with 0.1% Tween 20. Hoechst 33342 staining of larvae was carried out at room temperature for 10 min, followed by 3×10 min washes with PBS, 0.1% Tween 20. Larvae were then cleared by sequential incubation in increasing glycerol concentrations: 15% glycerol for 1 h at room temperature, 30% glycerol overnight at 4°C, 60% glycerol overnight at 4°C, 80% glycerol overnight at 4°C, before imaging by AiryScan confocal microscopy (Zeiss LSM 880). *Z*-stacks were obtained at intervals of 0.46 µm using the AiryScan SR mode.

### Antibodies

The primary antibody used was mouse α-GRA2 (#BIO.018.5, BIOTEM), a kind gift from Moritz Treeck, The Francis Crick Institute, London, UK. Secondary antibody used was goat α-mouse AF647 (A-21245, Invitrogen).

### 3D CLEM

Euthanized larvae were fixed overnight at 4°C in 4% formaldehyde (Taab Laboratories Equipment). Hoechst 33342 staining of larvae was carried out at room temperature for 10 min without permeabilization, and larvae were subsequently washed 3×10 min with 0.1 M phosphate buffer (PB). Larvae were embedded in 3% low-melt agarose in 35 mm glass-bottomed dishes. Larvae were covered in 0.1 M PB for high-resolution confocal microscopy (Zeiss LSM 710). Larvae were maintained in 1% formaldehyde in 0.1 M PB until further processing. The embedded larvae were sectioned using a Leica VT1000 S vibrating blade microtome (Leica Biosystems). Fifty-micrometer sections were collected and stored in 0.1 M PB in a 24-well glass-bottomed plate (MatTek). The sections were imaged again using a Zeiss Invert 710 LSM confocal (Carl Zeiss AG) and a 20× Ph2 objective. The sections containing *Toxoplasma* were then processed following the method of the National Center for Microscopy and Imaging Research (Deerink et al., 2010). In brief, they were post-fixed in 2.5% (v/v) glutaraldehyde/4% (v/v) formaldehyde in 0.1 M PB for 30 min at room temperature, stained in 2% osmium tetroxide/1.5% potassium ferricyanide for 1 h on ice and incubated in 1% w/v thiocarbohydrazide for 20 min, before a second staining with 2% osmium tetroxide and incubation overnight in 1% aqueous uranyl acetate at 4°C. Sections were stained with Walton's lead aspartate for 30 min at 60°C and dehydrated stepwise through an ethanol series on ice, incubated in a 1:1 propylene oxide/Durcupan resin mixture and embedded in Durcupan ACM^®^ resin according to the manufacturer's instructions (Sigma-Aldrich). Blocks were trimmed to a small trapezoid, excised from the resin block and attached to a SBF SEM specimen pin using conductive epoxy resin (Circuitworks CW2400).

#### SBF SEM

Prior to commencement of a SBF SEM imaging run, the sample was coated with a 2 nm layer of platinum to further enhance conductivity using a Q150R S sputter coater (Quorum Technologies). SBF SEM data were collected using a 3View2XP (Gatan) attached to a Sigma VP SEM (Zeiss). Inverted backscattered electron images were acquired through the entire extent of the ROI. For each 50 nm slice, a low-resolution overview image (pixel size of ∼50 nm using a 1.5 µs dwell time) and several high-resolution images of the different regions of interest (indicated magnification ∼5000×, pixel size of 6-7 nm using a 1.5 µs dwell time) were acquired. The overview image was used to relocate the ROI defined by the confocal images of the sections. The SEM was operated in variable pressure mode at 5 Pa. The 30 µm aperture was used, at an accelerating voltage of 2 kV. Typically, between 300 and 1000 slices were necessary for an entire ROI. As data were collected in variable pressure mode, only minor adjustments in image alignment were needed, particularly where the field of view was altered in order to track the cell of interest.

#### ssTEM

Prior to ssTEM, the sample was imaged by SBF SEM using low-resolution overview images (100 nm slices, 2 kV, pixel size of 33 nm, 2 µs dwell time, high vacuum with focal charge compensation on at 50%) to relocate the ROI defined by the confocal images. After SBF SEM, the sample was serial sectioned using a UC7 ultramicrotome (Leica Microsystems) and consecutive 70 nm sections were picked up on Formvar-coated 2 mm slot copper grids (Gilder Grids). Then, 143 consecutive sections containing 11 *Toxoplasma* were viewed at 440×, 890× and 6800× (plus some selected areas at 18,500×) using a 120 kV Tecnai G2 Spirit transmission electron microscope (Thermo Fisher Scientific), and images were captured using a OneView UltraScan^®^ 4000 camera and GMS3 software (Gatan). The three *Toxoplasma* shown in Fig. 6 were imaged in their full volume (∼75 sections).

After sectioning for ssTEM, the sample was imaged again in the SBF SEM at low and high resolution (pixel size of 35 nm and 6 nm, 50 nm slices, 2 kV, 2 µs dwell time, high vacuum with focal charge compensation on at 50%), until the macrophage to be imaged by FIB SEM appeared.

All the ssTEM and SBF SEM images were converted to tiff in Digital Micrograph or GMS3 (Gatan), and tiff stacks were automatically aligned using TrakEM2, a Fiji framework plug-in (Cardona et al., 2012). For ssTEM stacks of images, misaligned images were in addition manually aligned to the neighboring sections. Manual segmentations were performed in TrakEM2. For Figs 2A and 6A, labels were exported as tiff for visualization in 3D in ClearVolume, a Fiji plug-in (Royer et al., 2015). For Fig. S5B, they were exported as Amira labels for visualization in 3D in Amira Software (Thermo Fisher Scientific).

#### FIB SEM

FIB SEM data were collected using a Crossbeam 540 FIB SEM with Atlas 5 for 3D tomography acquisition (Zeiss, Cambridge). After image acquisition for SBF SEM and ssTEM was completed, the sample was further sputter coated with a 10 nm layer of platinum.

The ROI was relocated by briefly imaging through the platinum coating at an accelerating voltage of 20 kV and correlating to previously acquired SBF SEM and fluorescence microscopy images. The final ROI and milling orientation were targeted in order to preserve the majority of the sample whilst enclosing the entire cell of interest. On completion of preparation for milling and tracking, images were acquired at 5 nm isotropic resolution throughout the ROI, using a 4.2 µs dwell time. During acquisition, the SEM was operated at an accelerating voltage of 1.5 kV with 1.5 nA current. The EsB detector was used with a grid voltage of 1200 V. Ion beam milling was performed at an accelerating voltage of 30 kV and current of 700 pA.

The final dataset was acquired in two sessions; in order to correct for uneven milled slice thickness in the initial phase of the second session, the first 100 slices were aligned and interpolated to output a per-slice thickness of 5 nm (Atlas 5), prior to reinsertion into the dataset of ∼24.1 µm×17.8 µm×20.2 µm (8651 µm^3^).

After initial registration (template matching by normalized cross correlation; Fiji, https://sites.google.com/site/qingzongtseng/template-matching-ij-plugin), the images were batch processed to suppress noise, and enhance sharpness and contrast [(1) Gaussian blur 0.8 pixel radius; (2) smart sharpening with highlights suppressed: radius 10 pixels, strength 60%, then radius 1.2 pixels, strength 150%; (3) application of a medium contrast curve; (4) 8-bit grayscale conversion; Adobe Photoshop 2020]. Finally, to fine tune image registration, the alignment to median smoothed template method was applied (AMST; Hennies et al., 2020). Subregions comprising the macrophage and individual *Toxoplasma* were then cropped out from the final volume.

For Fig. 7A, image stacks from confocal microscopy and FIB SEM were manually aligned to each other using the BigWarp plugin of the Fiji framework (Bogovic et al., 2016), with the FIB SEM stack set as ‘target’ and the confocal stack as ‘moving’ dataset. ‘Landmark mode’ was used to add 19 pairs of corresponding points within both datasets throughout the whole volume of the cell. An affine transformation was applied to the confocal dataset.

Movies 2, 6 and 7 were generated in Fiji, Movie 4 was generated in Amira, and all were compressed in Quick Time Pro with the H.264 encoder.

### Measurement of leukocyte recruitment to the site of infection

Anesthetized larvae were imaged at 0, 6 and 24 hpi by fluorescent stereomicroscopy (Leica M205FA). Fifteen *z*-slices at 8.55 µm intervals covering 128 µm were taken at 130× magnification. Images were further analyzed using Fiji software.

### Metronidazole-targeted macrophage depletion

For macrophage ablation, the nitroreductase/metronidazole ablation system was utilized (Curado et al., 2008). Metronidazole is converted by nitroreductase into a cytotoxic metabolite. Therefore, expression of nitroreductase-mCherry (*nfsB.*mCherry) in the macrophage population in the transgenic line Tg(*mpeg1*:*Gal4-FF*)*^gl25^/*Tg(*UAS-E1b*:*nfsB*.mCherry)*^c264^* allows for the specific ablation of macrophages upon metronidazole treatment. Dechorionated 2 dpf TraNac-Tg(*mpeg1*:*Gal4-FF*)*^gl25^/*Tg(*UAS-E1b*:*nfsB*.mCherry)*^c264^* larvae were placed in embryo medium supplemented with metronidazole (10 mM; Sigma-Aldrich), 1% DMSO. Larvae were then placed in fresh 10 mM metronidazole solution at 33°C post-infection. Control-treated larvae were maintained in embryo medium supplemented with 1% DMSO.

### Statistical analysis

Significance testing was performed using unpaired Student's *t*-test, one-way ANOVA or two-way ANOVA (repeated measures with Sidak's/Tukey's multiple comparisons test). For data that do not conform to the assumptions of parametric statistics, Chi-square or Kruskal–Wallis (with Dunn's multiple comparisons) tests were used.

## Supplementary Material

Supplementary information

## References

[DMM043091C1] Ajzenberg D., Cogné N., Paris L., Bessières M.-H., Thulliez P., Filisetti D., Pelloux H., Marty P. and Darde M. L. (2002). Genotype of 86 *Toxoplasma gondii* isolates associated with human congenital toxoplasmosis, and correlation with clinical findings. *J. Infect. Dis.* 186, 684-689. 10.1086/342663 12195356

[DMM043091C2] Ajzenberg D., Yera H., Marty P., Paris L., Dalle F., Menotti J., Aubert D., Franck J., Bessières M.-H., Quinio D. et al. (2009). Genotype of 88 *Toxoplasma gondii* isolates associated with toxoplasmosis in immunocompromised patients and correlation with clinical findings. *J. Infect. Dis.* 199, 1155-1167. 10.1086/597477 19265484

[DMM043091C3] Andrade R. M., Portillo J. A. C., Wessendarp M. and Subauste C. S. (2005). CD40 signaling in macrophages induces activity against an intracellular pathogen independently of gamma interferon and reactive nitrogen intermediates. *Infect. Immun.* 73, 3115-3123. 10.1128/IAI.73.5.3115-3123.2005 15845519 PMC1087328

[DMM043091C4] Black M. W. and Boothroyd J. C. (2000). Lytic cycle of *Toxoplasma gondii*. *Microbiol. Mol. Biol. Rev.* 64, 607-623. 10.1128/MMBR.64.3.607-623.2000 10974128 PMC99006

[DMM043091C5] Bogovic J. A., Hanslovsky P., Wong A. and Saalfeld S. (2016). Robust registration of calcium images by learned contrast synthesis. *ISBI* 1123-1126. 10.1109/ISBI.2016.7493463

[DMM043091C6] Cabral C. M., Tuladhar S., Dietrich H. K., Nguyen E., MacDonald W. R., Trivedi T., Devineni A. and Koshy A. A. (2016). Neurons are the primary target cell for the brain-tropic intracellular parasite *Toxoplasma gondii*. *PLoS Pathog.* 12, e1005447 10.1371/journal.ppat.1005447 26895155 PMC4760770

[DMM043091C7] Cardona A., Saalfeld S., Schindelin J., Arganda-Carreras I., Preibisch S., Longair M., Tomancak P., Hartenstein V. and Douglas R. J. (2012). TrakEM2 software for neural circuit reconstruction. *PLoS ONE* 7, e38011 10.1371/journal.pone.0038011 22723842 PMC3378562

[DMM043091C8] Channon J. Y., Seguin R. M. and Kasper L. H. (2000). Differential infectivity and division of *Toxoplasma gondii* in human peripheral blood leukocytes. *Infect. Immun.* 68, 4822-4826. 10.1128/IAI.68.8.4822-4826.2000 10899898 PMC98447

[DMM043091C9] Clough B. and Frickel E. M. (2017). The *Toxoplasma* parasitophorous vacuole: an evolving host-parasite frontier. *Trends Parasitol.* 33, 473-488. 10.1016/j.pt.2017.02.007 28330745

[DMM043091C10] Coombes J. L., Charsar B. A., Han S.-J., Halkias J., Chan S. W., Koshy A. A., Striepen B. and Robey E. A. (2013). Motile invaded neutrophils in the small intestine of *Toxoplasma gondii*-infected mice reveal a potential mechanism for parasite spread. *Proc. Natl. Acad. Sci. USA* 110, E1913-E1922. 10.1073/pnas.1220272110 23650399 PMC3666704

[DMM043091C11] Courret N., Darche S., Sonigo P., Milon G., Buzoni-Gâtel D. and Tardieux I. (2006). CD11c- and CD11b-expressing mouse leukocytes transport single *Toxoplasma gondii t*achyzoites to the brain. *Blood* 107, 309-316. 10.1182/blood-2005-02-0666 16051744 PMC1895351

[DMM043091C12] Curado S., Stainier D. Y. R. and Anderson R. M. (2008). Nitroreductase-mediated cell/tissue ablation in zebrafish: a spatially and temporally controlled ablation method with applications in developmental and regeneration studies. *Nat. Protoc.* 3, 948-954. 10.1038/nprot.2008.58 18536643 PMC2705989

[DMM043091C13] Deerink T. J., Bushong E. A. and Ellisman M. H (2010). NCMIR methods for 3D EM: A new protocol for preparation for biological specimens for serial blockface scanning electron microscopy. https://ncmir.ucsd.edu/sbem-protocol.

[DMM043091C14] Del Rio L., Bennouna S., Salinas J. and Denkers E. Y. (2001). CXCR2 deficiency confers impaired neutrophil recruitment and increased susceptibility during *Toxoplasma gondii* infection. *J. Immunol.* 167, 6503-6509. 10.4049/jimmunol.167.11.6503 11714818

[DMM043091C15] Denkers E. Y., Schneider A. G., Cohen S. B. and Butcher B. A. (2012). Phagocyte responses to protozoan infection and how *Toxoplasma gondii* meets the challenge. *PLoS Pathog.* 8, e1002794 10.1371/journal.ppat.1002794 22876173 PMC3410898

[DMM043091C16] Dóró E., Jacobs S. H., Hammond F. R., Schipper H., Pieters R. P., Carrington M., Wiegertjes G. F. and Forlenza M. (2019). Visualizing trypanosomes in a vertebrate host reveals novel swimming behaviours, adaptations and attachment mechanisms. *Elife* 8, e48388 10.7554/eLife.48388 31547905 PMC6759355

[DMM043091C17] Dunay I. R., Damatta R. A., Fux B., Presti R., Greco S., Colonna M. and Sibley L. D. (2008). Gr1+ inflammatory monocytes are required for mucosal resistance to the pathogen *Toxoplasma gondii*. *Immunity* 29, 306-317. 10.1016/j.immuni.2008.05.019 18691912 PMC2605393

[DMM043091C18] Ferguson D. J. and Hutchison W. M. (1987). The host-parasite relationship of *Toxoplasma gondii* in the brains of chronically infected mice. *Virchows Arch. A Pathol. Anat. Histopathol.* 411, 39-43. 10.1007/BF00734512 3107207

[DMM043091C19] Fisch D., Clough B., Domart M.-C., Encheva V., Bando H., Snijders A. P., Collinson L. M., Yamamoto M., Shenoy A. R., and Frickel E.-M. (2020). Human GBP1 differentially targets *Salmonella* and *Toxoplasma* to license recognition of microbial ligands and caspase-mediated death. *bioRxiv*. 10.1101/792804 PMC7435695 32783936

[DMM043091C20] Gazzinelli R. T., Mendonça-Neto R., Lilue J., Howard J. and Sher A. (2014). Innate resistance against *Toxoplasma gondii*: an evolutionary tale of mice, cats, and men. *Cell Host Microbe* 15, 132-138. 10.1016/j.chom.2014.01.004 24528860 PMC4006104

[DMM043091C21] Gomes M. C. and Mostowy S. (2020). The case for modelling human infection in zebrafish. *Trends Microbiol.* 28, 10-18. 10.1016/j.tim.2019.08.005 31604611

[DMM043091C22] Gregg B., Taylor B. C., John B., Tait-Wojno E. D., Girgis N. M., Miller N., Wagage S., Roos D. S. and Hunter C. A. (2013). Replication and distribution of *Toxoplasma gondii* in the small intestine after oral infection with tissue cysts. *Infect. Immun.* 81, 1635-1643. 10.1128/IAI.01126-12 23460516 PMC3647985

[DMM043091C23] Haldar A. K., Foltz C., Finethy R., Piro A. S., Feeley E. M., Pilla-Moffett D. M., Komatsu M., Frickel E. M. and Coers J. (2015). Ubiquitin systems mark pathogen-containing vacuoles as targets for host defense by guanylate binding proteins. *Proc. Natl. Acad. Sci. USA* 112, E5628-E5637. 10.1073/pnas.1515966112 26417105 PMC4611635

[DMM043091C24] Harker K. S., Ueno N. and Lodoen M. B. (2015). Toxoplasma gondii dissemination: a parasite's journey through the infected host. *Parasite Immunol.* 37, 141-149. 10.1111/pim.12163 25408224

[DMM043091C25] Hennies J., Lleti J. M. S., Schieber N. L., Templin R. M., Steyer A. M. and Schwab Y. (2020). AMST: alignment to median smoothed template for focused ion beam scanning electron microscopy image stacks. *Sci. Rep.* 10, 2004 10.1038/s41598-020-58736-7 32029771 PMC7004979

[DMM043091C26] Howe D. K. and Sibley L. D. (1995). Toxoplasma gondii comprises three clonal lineages: correlation of parasite genotype with human disease. *J. Infect. Dis.* 172, 1561-1566. 10.1093/infdis/172.6.1561 7594717

[DMM043091C27] Igawa D., Sakai M. and Savan R. (2006). An unexpected discovery of two interferon gamma-like genes along with interleukin (IL)-22 and −26 from teleost: IL-22 and −26 genes have been described for the first time outside mammals. *Mol. Immunol.* 43, 999-1009. 10.1016/j.molimm.2005.05.009 16005068

[DMM043091C28] Khan A., Behnke M. S., Dunay I. R., White M. W. and Sibley L. D. (2009). Phenotypic and gene expression changes among clonal type I strains of *Toxoplasma gondii*. *Eukaryot. Cell* 8, 1828-1836. 10.1128/EC.00150-09 19801420 PMC2794221

[DMM043091C29] Krauss J., Astrinidis P., Astrinides P., Frohnhofer H. G., Walderich B. and Nusslein-Volhard C. (2013). transparent, a gene affecting stripe formation in Zebrafish, encodes the mitochondrial protein Mpv17 that is required for iridophore survival. *Biol. Open* 2, 703-710. 10.1242/bio.20135132 23862018 PMC3711038

[DMM043091C30] López-Muñoz A., Roca F. J., Meseguer J. and Mulero V. (2009). New insights into the evolution of IFNs: zebrafish group II IFNs induce a rapid and transient expression of IFN-dependent genes and display powerful antiviral activities. *J. Immunol.* 182, 3440-3449. 10.4049/jimmunol.0802528 19265122

[DMM043091C31] López-Muñoz A., Sepulcre M. P., Roca F. J., Figueras A., Meseguer J. and Mulero V. (2011). Evolutionary conserved pro-inflammatory and antigen presentation functions of zebrafish IFNgamma revealed by transcriptomic and functional analysis. *Mol. Immunol.* 48, 1073-1083. 10.1016/j.molimm.2011.01.015 21354627

[DMM043091C32] Martens S., Parvanova I., Zerrahn J., Griffiths G., Schell G., Reichmann G. and Howard J. C. (2005). Disruption of *Toxoplasma gondii* parasitophorous vacuoles by the mouse p47-resistance GTPases. *PLoS Pathog.* 1, e24 10.1371/journal.ppat.0010024 16304607 PMC1287907

[DMM043091C33] Mazon Moya M. J., Willis A. R., Torraca V., Boucontet L., Shenoy A. R., Colucci-Guyon E. and Mostowy S. (2017). Septins restrict inflammation and protect zebrafish larvae from *Shigella* infection. *PLoS Pathog.* 13, e1006467 10.1371/journal.ppat.1006467 28650995 PMC5507465

[DMM043091C34] Mercier C., Dubremetz J.-F., Rauscher B., Lecordier L., Sibley L. D. and Cesbron-Delauw M.-F. (2002). Biogenesis of nanotubular network in *Toxoplasma* parasitophorous vacuole induced by parasite proteins. *Mol. Biol. Cell* 13, 2397-2409. 10.1091/mbc.e02-01-0021 12134078 PMC117322

[DMM043091C35] Mordue D. G. and Sibley L. D. (2003). A novel population of Gr-1 +-activated macrophages induced during acute toxoplasmosis. *J. Leukoc. Biol.* 74, 1015-1025. 10.1189/jlb.0403164 12972511

[DMM043091C36] Murray H. W. and Cohn Z. A. (1979). Macrophage oxygen-dependent antimicrobial activity. I. Susceptibility of *Toxoplasma gondii* to oxygen intermediates. *J. Exp. Med.* 150, 938-949. 10.1084/jem.150.4.938 92521 PMC2185675

[DMM043091C37] Murray H. W., Juangbhanich C. W., Nathan C. F. and Cohn Z. A. (1979). Macrophage oxygen-dependent antimicrobial activity. II. The role of oxygen intermediates. *J. Exp. Med.* 150, 950-964. 10.1084/jem.150.4.950 512587 PMC2185678

[DMM043091C38] Pappas G., Roussos N. and Falagas M. E. (2009). Toxoplasmosis snapshots: global status of *Toxoplasma gondii* seroprevalence and implications for pregnancy and congenital toxoplasmosis. *Int. J. Parasitol.* 39, 1385-1394. 10.1016/j.ijpara.2009.04.003 19433092

[DMM043091C39] Park H.-C., Kim C.-H., Bae Y.-K., Yeo S.-Y., Kim S.-H., Hong S.-K., Shin J., Yoo K.-W., Hibi M., Hirano T. et al. (2000). Analysis of upstream elements in the HuC promoter leads to the establishment of transgenic zebrafish with fluorescent neurons. *Dev. Biol.* 227, 279-293. 10.1006/dbio.2000.9898 11071755

[DMM043091C40] Pernas L., Adomako-Ankomah Y., Shastri A. J., Ewald S. E., Treeck M., Boyle J. P. and Boothroyd J. C. (2014). Toxoplasma effector MAF1 mediates recruitment of host mitochondria and impacts the host response. *PLoS Biol.* 12, e1001845 10.1371/journal.pbio.1001845 24781109 PMC4004538

[DMM043091C41] Pittman K. J. and Knoll L. J. (2015). Long-term relationships: the complicated Interplay between the host and the developmental stages of *Toxoplasma gondii* during acute and chronic infections. *Microbiol. Mol. Bio. Rev.* 79, 387-401. 10.1128/MMBR.00027-15 26335719 PMC4557073

[DMM043091C42] Renshaw S. A. and Trede N. S. (2012). A model 450 million years in the making: zebrafish and vertebrate immunity. *Dis. Model. Mech.* 5, 38-47. 10.1242/dmm.007138 22228790 PMC3255542

[DMM043091C43] Robben P. M., Laregina M., Kuziel W. A. and Sibley L. D. (2005). Recruitment of Gr-1+ monocytes is essential for control of acute toxoplasmosis. *J. Exp. Med.* 201, 1761-1769. 10.1084/jem.20050054 15928200 PMC2213275

[DMM043091C44] Royer L. A., Weigert M., Günther U., Maghelli N., Jug F., Sbalzarini I. F. and Myers E. W. (2015). ClearVolume: open-source live 3D visualization for light-sheet microscopy. *Nat. Methods* 12, 480-481. 10.1038/nmeth.3372 26020498

[DMM043091C45] Saeij J. P. and Frickel E.-M. (2017). Exposing *Toxoplasma gondii* hiding inside the vacuole: a role for GBPs, autophagy and host cell death. *Curr. Opin. Microbiol.* 40, 72-80. 10.1016/j.mib.2017.10.021 29141239 PMC7004510

[DMM043091C46] Saeij J. P., Boyle J. P. and Boothroyd J. C. (2005). Differences among the three major strains of *Toxoplasma gondii* and their specific interactions with the infected host. *Trends Parasitol.* 21, 476-481. 10.1016/j.pt.2005.08.001 16098810

[DMM043091C47] Safronova A., Araujo A., Camanzo E. T., Moon T. J., Elliott M. R., Beiting D. P. and Yarovinsky F. (2019). Alarmin S100A11 initiates a chemokine response to the human pathogen *Toxoplasma gondii*. *Nat. Immunol.* 20, 64-72. 10.1038/s41590-018-0250-8 30455460 PMC6291348

[DMM043091C48] Schindelin J., Arganda-Carreras I., Frise E., Kaynig V., Longair M., Pietzsch T., Preibisch S., Rueden C., Saalfeld S., Schmid B. et al. (2012). Fiji: an open-source platform for biological-image analysis. *Nat. Methods* 9, 676-682. 10.1038/nmeth.2019 22743772 PMC3855844

[DMM043091C49] Selleck E. M., Orchard R. C., Lassen K. G., Beatty W. L., Xavier R. J., Levine B., Virgin H. W. and Sibley L. D. (2015). A noncanonical autophagy pathway restricts *Toxoplasma gondii* growth in a strain-specific manner in IFN-gamma-activated human cells. *mBio* 6, e01157-e01115. 10.1128/mBio.01157-15 26350966 PMC4600106

[DMM043091C50] Sher A., Tosh K. and Jankovic D. (2017). Innate recognition of *Toxoplasma gondii* in humans involves a mechanism distinct from that utilized by rodents. *Cell. Mol. Immunol.* 14, 36-42. 10.1038/cmi.2016.12 27157497 PMC5214937

[DMM043091C51] Sibley L. D., Adams L. B., Fukutomi Y. and Krahenbuhl J. L. (1991). Tumor necrosis factor-alpha triggers antitoxoplasmal activity of IFN-gamma primed macrophages. *J. Immunol.* 147, 2340-2345. 1918966

[DMM043091C52] Sieger D., Stein C., Neifer D., van der Sar A. M. and Leptin M. (2009). The role of gamma interferon in innate immunity in the zebrafish embryo. *Dis. Model. Mech.* 2, 571-581. 10.1242/dmm.003509 19779068

[DMM043091C53] Szabo E. K. and Finney C. A. M. (2017). Toxoplasma gondii: one organism, multiple models. *Trends Parasitol.* 33, 113-127. 10.1016/j.pt.2016.11.007 27988095

[DMM043091C54] Torraca V. and Mostowy S. (2018). Zebrafish infection: from pathogenesis to cell biology. *Trends Cell Biol.* 28, 143-156. 10.1016/j.tcb.2017.10.002 29173800 PMC5777827

[DMM043091C55] Tosh K. W., Mittereder L., Bonne-Annee S., Hieny S., Nutman T. B., Singer S. M., Sher A. and Jankovic D. (2016). The IL-12 response of primary human dendritic cells and monocytes to *Toxoplasma gondii* is stimulated by phagocytosis of live parasites rather than host cell invasion. *J. Immunol.* 196, 345-356. 10.4049/jimmunol.1501558 26597011 PMC4685007

[DMM043091C64] Vladimirov N., Mu Y., Kawashima T., Bennett D. V., Yang C. T., Looger L. L., Keller P. J., Freeman J., and Ahrens M. B. (2014). Light-sheet functional imaging in fictively behaving zebrafish. *Nat. Methods* 11, 883-884. 10.1038/nmeth.3040 25068735

[DMM043091C56] Westerfield M. (2007). *The Zebrafish Book. A Guide for the Laboratory use of Zebrafish (Danio rerio)*. Eugene: University of Oregon Press.

[DMM043091C57] Yakimovich A., Huttunen M., Samolei J., Clough B., Yoshida N., Mostowy S., Frickel E. M. and Mercer J (2019). Mimicry embedding for efficient capsule network weights transfer in 3D biomedical micrographs. *bioRxiv* 10.1101/820076

[DMM043091C58] Yamamoto M., Okuyama M., Ma J. S., Kimura T., Kamiyama N., Saiga H., Ohshima J., Sasai M., Kayama H., Okamoto T. et al. (2012). A cluster of interferon-gamma-inducible p65 GTPases plays a critical role in host defense against *Toxoplasma gondii*. *Immunity* 37, 302-313. 10.1016/j.immuni.2012.06.009 22795875

[DMM043091C59] Yang N., Farrell A., Niedelman W., Melo M., Lu D., Julien L., Marth G. T., Gubbels M.-J. and Saeij J. P. (2013). Genetic basis for phenotypic differences between different *Toxoplasma gondii* type I strains. *BMC Genomics* 14, 467 10.1186/1471-2164-14-467 23837824 PMC3710486

[DMM043091C60] Yarovinsky F., Hieny S. and Sher A. (2008). Recognition of *Toxoplasma gondii* by TLR11 prevents parasite-induced immunopathology. *J. Immunol.* 181, 8478-8484. 10.4049/jimmunol.181.12.8478 19050265 PMC4809201

[DMM043091C61] Yoon S., Alnabulsi A., Wang T. Y., Lee P. T., Chen T. Y., Bird S., Zou J. and Secombes C. J. (2016). Analysis of interferon gamma protein expression in zebrafish (Danio rerio). *Fish Shellfish Immunol.* 57, 79-86. 10.1016/j.fsi.2016.08.023 27539703

[DMM043091C62] Yoshida N., Frickel E. M. and Mostowy S. (2017). Macrophage–microbe interactions: lessons from the zebrafish model. *Front. Immunol.* 8, 508 10.3389/fimmu.2017.01703 29250076 PMC5717010

[DMM043091C63] Zhao Z., Fux B., Goodwin M., Dunay I. R., Strong D., Miller B. C., Cadwell K., Delgado M. A., Ponpuak M., Green K. G. et al. (2008). Autophagosome-independent essential function for the autophagy protein Atg5 in cellular immunity to intracellular pathogens. *Cell Host Microbe* 4, 458-469. 10.1016/j.chom.2008.10.003 18996346 PMC2682425

