# Supplementary material for: The zebrafish as a novel model for the in vivo study of Toxoplasma gondii replication and interaction with macrophages
Source: Dis Model Mech. 2020 Jul 20;13(7):dmm043091. doi: 10.1242/dmm.043091 (PMC7390642; doi:10.1242/dmm.043091)
Supplement: Supplementary information [file dmm-13-043091-s1.pdf]

Supplementary Information

Supplementary Figure 1.

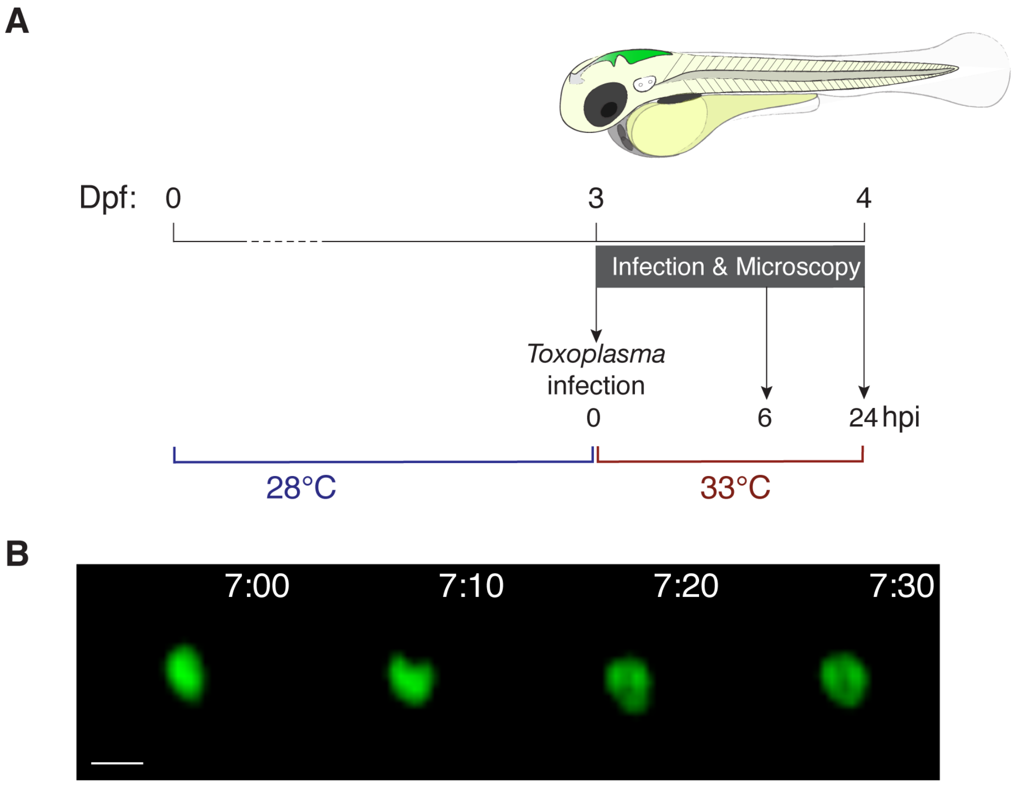

Disease Models & Mechanisms • Supplementary information

Figure S1. *Toxoplasma gondii* tachyzoites are intracellular and replicate in zebrafish, related to Figure 1. (A) Schematic of the infection model utilized with a cartoon of the zebrafish larva (3 dpf) highlighting the site of *Toxoplasma* tachyzoite injection in the hindbrain ventricle (HBV) in green. Infected larvae were maintained at 33°C post-injection and monitored up to 24 hours post-infection (hpi). (B) Representative frames extracted from *in vivo* widefield imaging of larvae injected with type I *Toxoplasma*-GFP (green). First frame at 7 hpi followed by three consecutive frames taken at 10 minute intervals. Showing a single Z-plane from 60 taken at 2 μm optical sections. Scale bar, 5 μm. See also Movie 1.

Supplementary Figure 2.

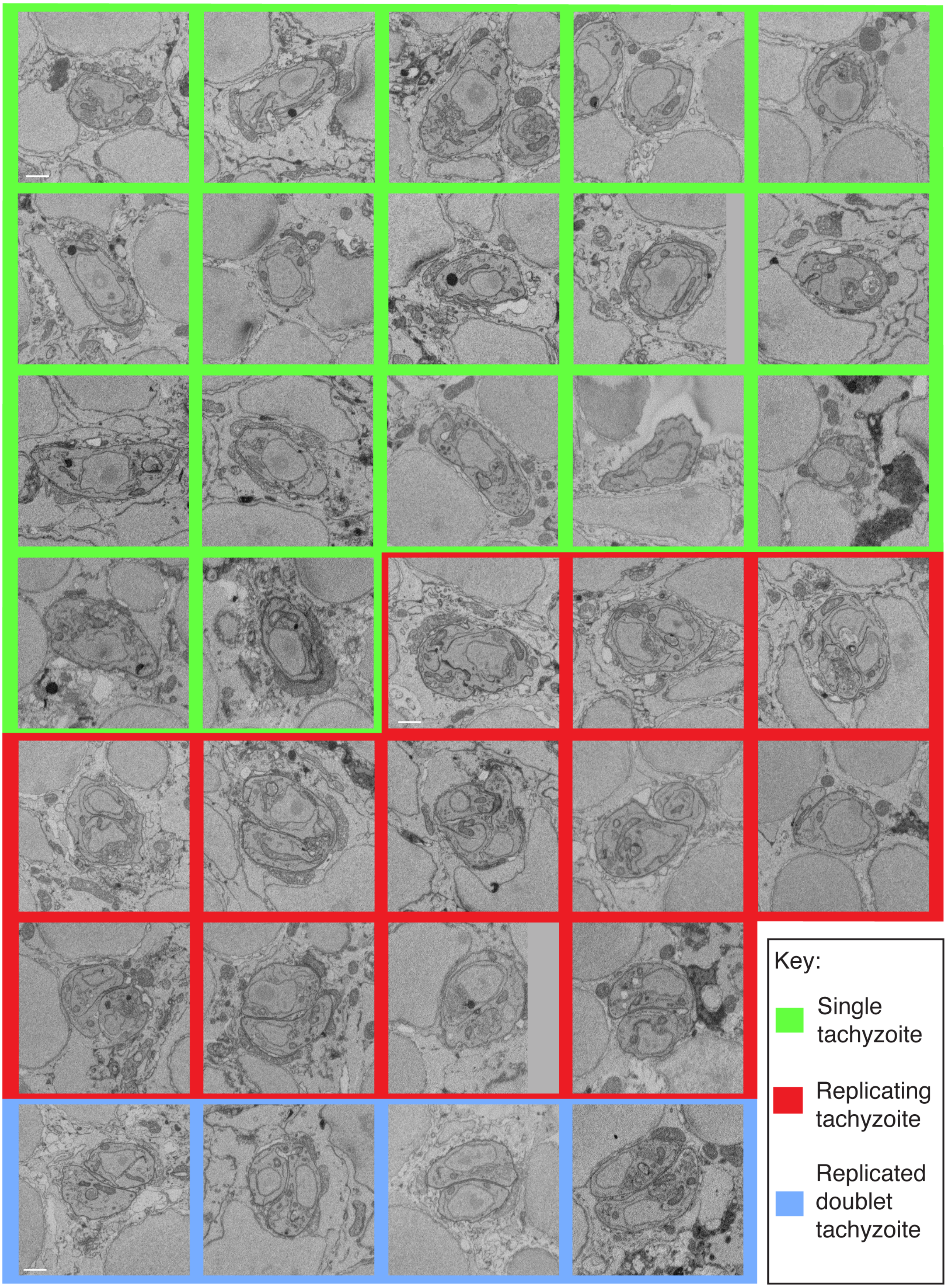

Disease Models & Mechanisms • Supplementary information

Figure S2. 3D CLEM of single, replicating and doublet tachyzoites in the zebrafish hindbrain, related to Figure 2. 3D CLEM of tachyzoites in the HBV of *mpeg1:G:U*:mCherry larvae infected with type I *Toxoplasma*-GFP at 6 hpi. Representative images of 33/36 *Toxoplasma* in zebrafish host cells extracted from stacks of consecutive 50 nm SBF SEM slices. *Toxoplasma* tachyzoites were imaged in their full volume to accurately determine their replicative stage. Single (green box), replicating (red box) and replicated doublet (blue box) tachyzoites were observed. Scale bar, 1 μm.

Disease Models & Mechanisms • Supplementary information

Supplementary Figure 3.

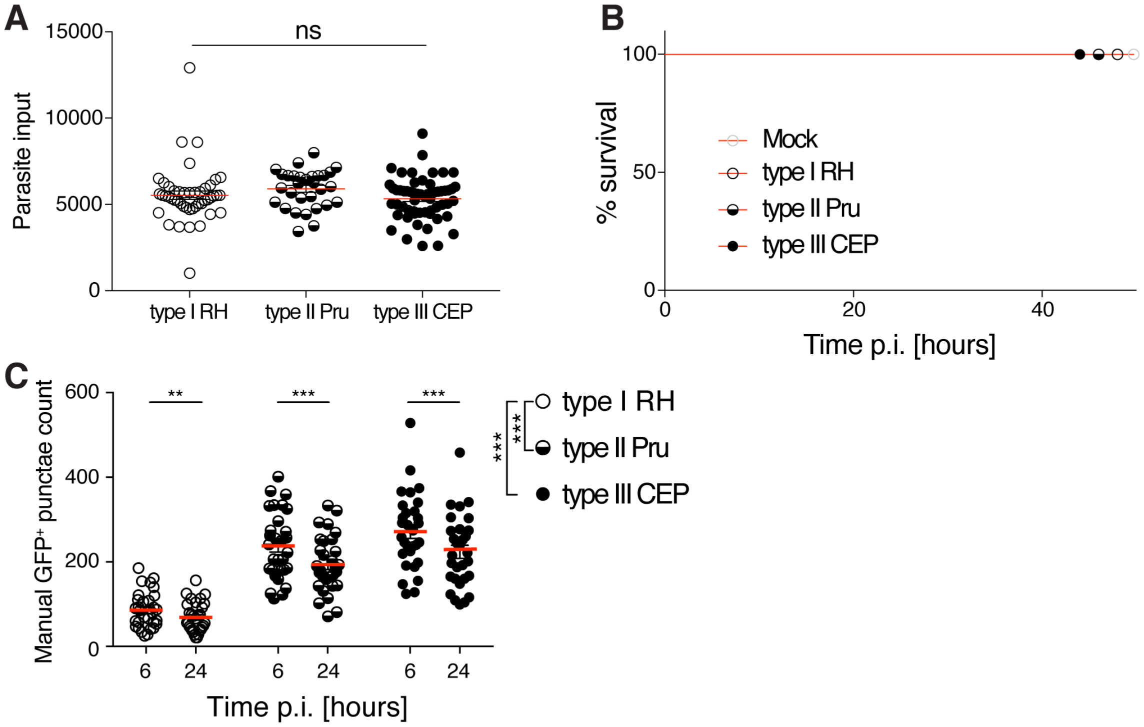

Disease Models & Mechanisms • Supplementary information

Figure S3. The zebrafish larvae model of acute *Toxoplasma gondii* infection is non-lethal, related to Figure 3. (A) Quantification of type I (RH, open circle), type II (Pru, semi-closed circle) or type III (CEP, closed circle) input dose of *Toxoplasma*-GFP using particle analysis of infected HBV images obtained by fluorescent stereomicroscopy at 0 hpi. Mean ± SEM shown. Significance calculated using one-way ANOVA with Tukey's multiple comparisons test, ns, $p>0.05$. (B) Survival curves of larvae injected with mock (human foreskin fibroblast lysate), type I (RH), type II (Pru) or type III (CEP) *Toxoplasma* tachyzoites. Pooled data from at least 3 independent experiments with at least 5 larvae per condition per experiment. (C) Manual enumeration of GFP-positive punctae in the HBV at 6 and 24 hpi of type I (RH, open circle), type II (Pru, semi-closed circle) or type III (CEP, closed circle) *Toxoplasma*-GFP. Mean ± SEM shown. Pooled data from at least 3 independent experiments with at least 5 larvae per condition per experiment. Significance calculated using 2-way ANOVA (repeated measures) with Sidak's multiple comparisons test, **, $p\leq0.01$, ***, $p\leq0.001$.

Disease Models & Mechanisms • Supplementary information

Supplementary Figure 4.

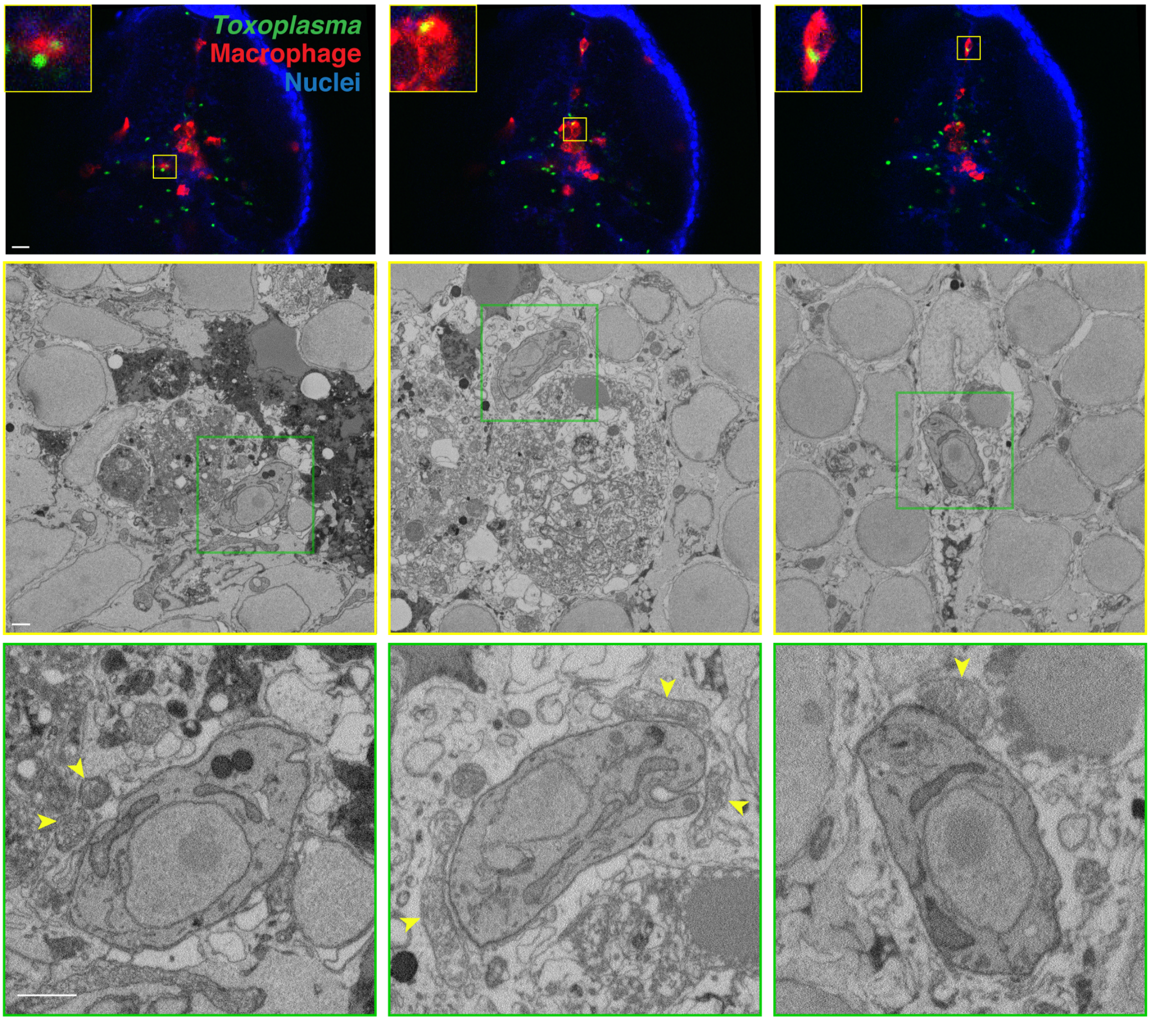

Disease Models & Mechanisms • Supplementary information

Figure S4. *Toxoplasma gondii* actively invade zebrafish macrophages. 3D CLEM of parasites inside macrophages in the HBV of *mpeg1:G/U:*mCherry (red) larvae infected with type I *Toxoplasma-GFP* (green) at 6 hpi stained with Hoechst 33342 (blue). Representative images extracted from 44 confocal Z-slices of a full section (top panels). The localizations of respective high-resolution SBF SEM images (middle panels) are denoted with yellow boxes. The localization of respective enlarged high-resolution SBF SEM images (bottom panels) of *Toxoplasma* are denoted with green boxes. Host mitochondrial recruitment to the parasitophorous vacuole indicated by yellow arrowheads. Scale bars, 20 μm (top panels) and 1 μm (middle and bottom panels).

Disease Models & Mechanisms • Supplementary information

Supplementary Figure 5.

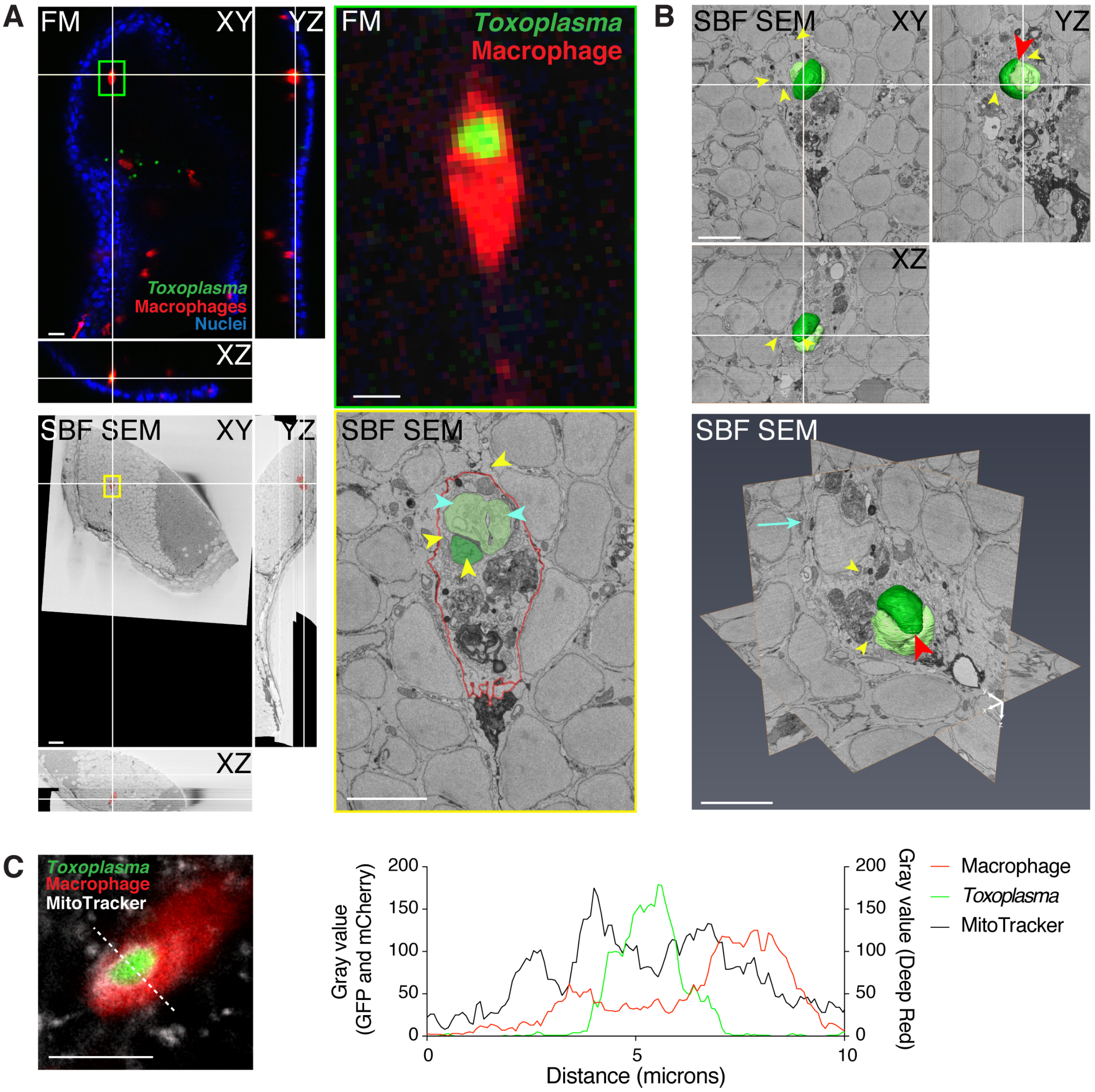

Disease Models & Mechanisms • Supplementary information

Figure S5. Replicative *Toxoplasma gondii* inside a zebrafish macrophage. (A) 3D CLEM of parasite replication inside a macrophage in the HBV of *mpeg1:G/U*:mCherry (red) larvae infected with type I *Toxoplasma*-GFP (green) at 6 hpi and stained with Hoechst 33342 (blue). Orthoslices of 44 confocal Z- slices of a full vibratome section (FM, left top panel) and of 1662 consecutive 50 nm SBF SEM slices from a subregion of it (left bottom panel). Color boxes show localization of the confocal (right top, green) and high-resolution SBF SEM images (right bottom, yellow). The two replicating *Toxoplasma* (green and light green) and plasma membrane of the macrophage (red) were manually segmented. Blue arrowheads in the light green *Toxoplasma* indicates two nuclei. Host mitochondrial recruitment to the PV indicated by yellow arrowheads. Scale bars, 20 μm (left) and 5 μm (right). (B) Orthoslices (top panel) and 3D view (bottom panel) of the 3D model of the two segmented type I *Toxoplasma* shown in (A) overlaid on 373 consecutive 50 nm SBF SEM slices. Area where the two *Toxoplasma* are still joined indicated by the red arrowhead, nucleus of the macrophage by the blue arrow, host mitochondrial recruitment to the PV by yellow arrowheads. Scale bars, 5 μm. See also Movie 4. (C) Representative image extracted from a Z-stack from confocal imaging of live *mpeg1:G/U*:mCherry (red) larvae infected in the HBV with type I *Toxoplasma*-GFP (green) and stained with MitoTracker (grey) at 6 hpi and the fluorescent intensity profile of a parasite exhibiting host mitochondrial association within a macrophage. Image was smoothened post-acquisition by applying gaussian blur 0.75 pixel radius. Scale bar, 10 μm.

Disease Models & Mechanisms • Supplementary information

Supplementary Figure 6.

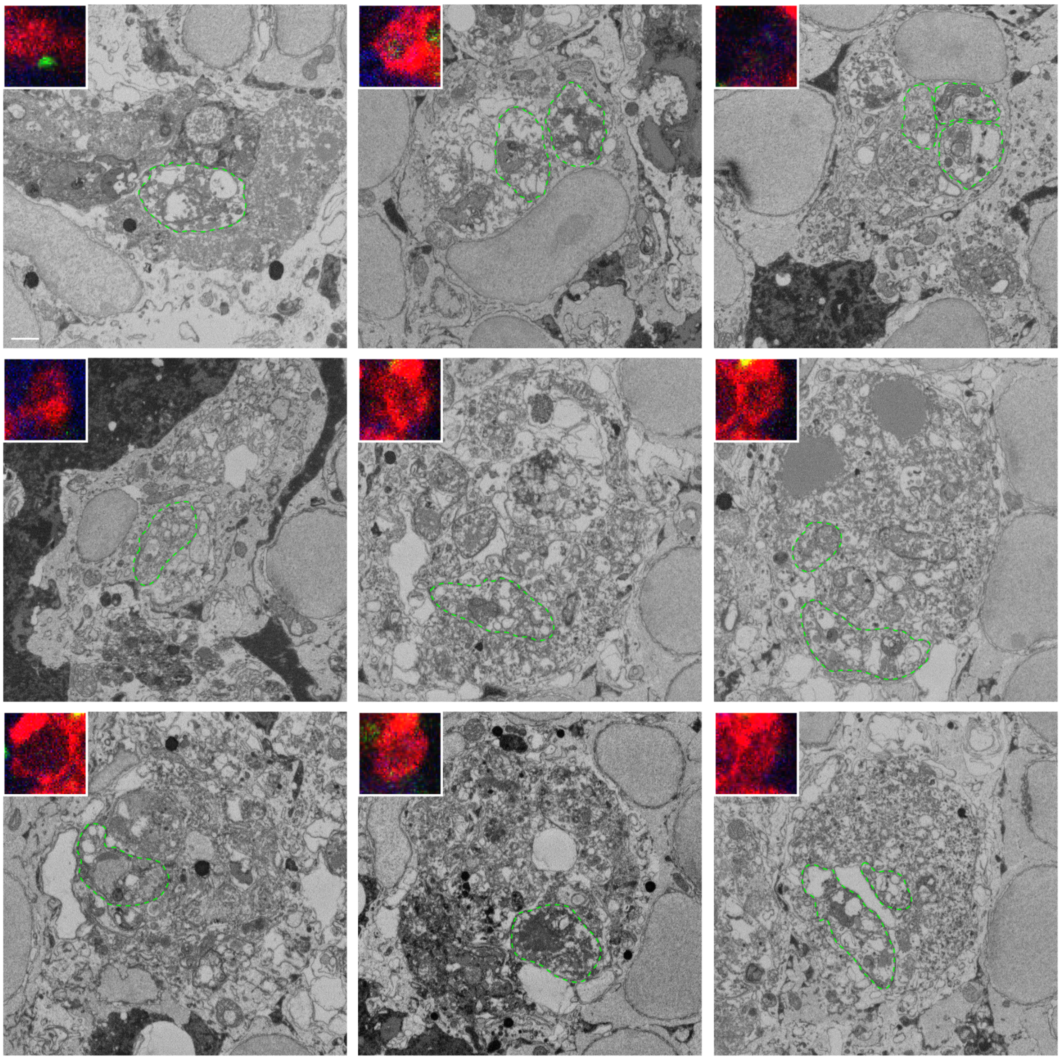

Figure S6. *Toxoplasma gondii* is degraded upon phagocytosis by zebrafish macrophages, related to Figure 5. 3D CLEM of putative dead tachyzoites in the HBV of *mpeg1:G/U:*mCherry (red) larvae infected with type I *Toxoplasma*-GFP (green) at 6 hpi and stained with Hoechst 33342 (blue). Representative images extracted from confocal Z-stacks of a full section (inset) and from Z-stacks of 50 nm SBF SEM slices of a segment of it. Only the first parasite (top left) showed GFP fluorescence. Putative dead parasites indicated by green dashed outline. Scale bar, 1 μm.

Disease Models & Mechanisms • Supplementary information

Supplementary Figure 7.

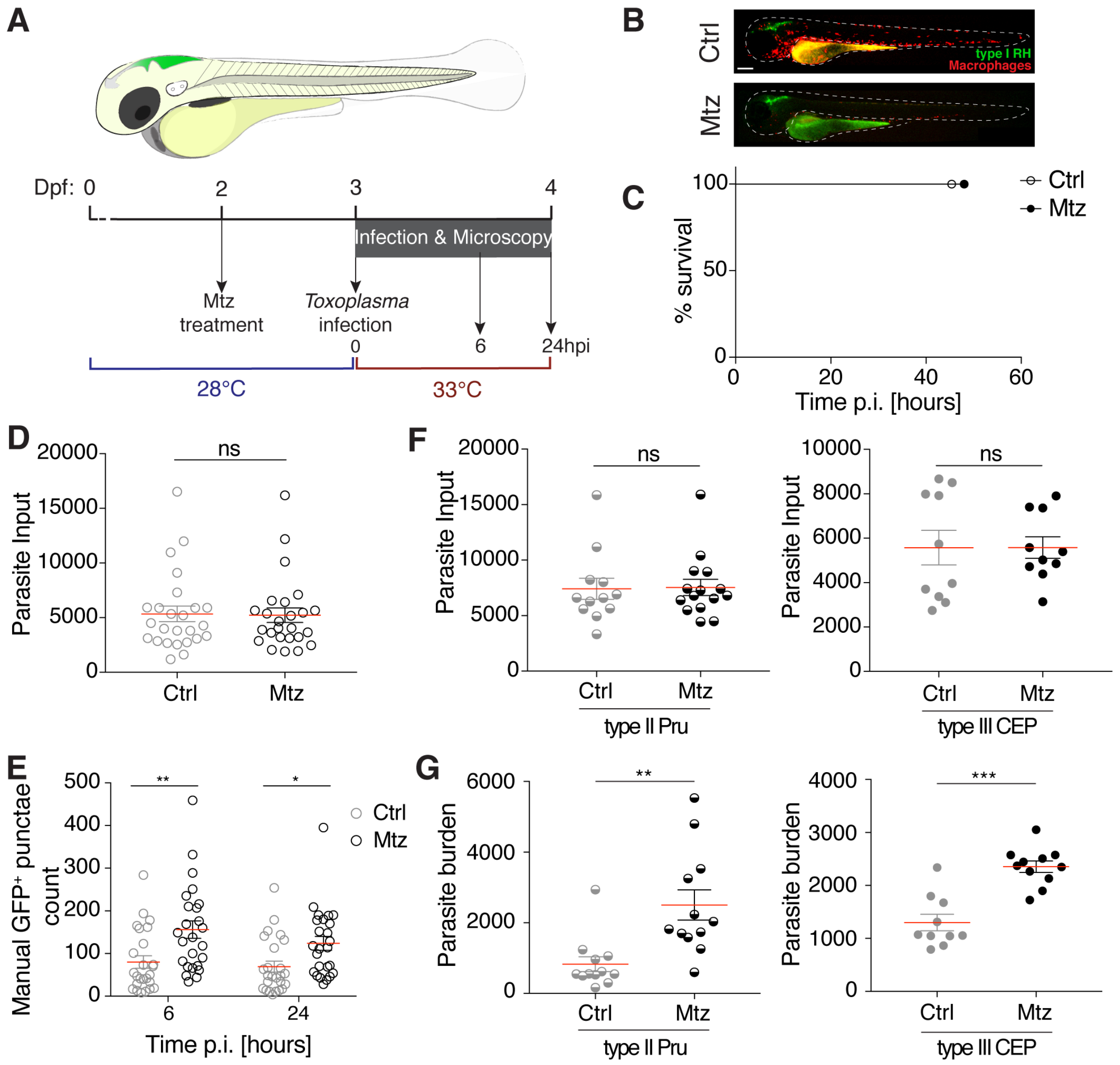

Figure S7. *Toxoplasma gondii* is controlled by zebrafish macrophages, related to Figure 8. (A) Schematic of the infection model utilized with a cartoon of the zebrafish larva (3 dpf) highlighting the site of *Toxoplasma* tachyzoite injection in the HBV in green. Prior to infection, larvae were pre-treated from 2 dpf with DMSO (control, Ctrl) or metronidazole (Mtz). Infected larvae were maintained at 33°C post-infection and monitored up to 24 hpi. (B) Representative images of Ctrl or Mtz treated *mpeg1:G/U*:mCherry larvae (red) injected with type I *Toxoplasma*-GFP (green) at 0 hpi. Scale bar, 200 μm. (C) Survival curves of Ctrl (open circles) or macrophage-ablated (Mtz-treated *mpeg1:G/U*:mCherry, closed circles) larvae infected in the HBV with type I *Toxoplasma*-GFP. Pooled data from 3 independent experiments with at least 7 larvae per condition per experiment. (D) Quantification of input dose of type I *Toxoplasma*-GFP in Ctrl (grey, open circle) or macrophage-ablated (Mtz; black, open circle) larvae using particle analysis of infected HBV images obtained by fluorescent stereomicroscopy at 0 hpi. Mean ± SEM shown. Significance calculated using unpaired t-test, ns, $p>0.05$. (E) Manual enumeration GFP-positive punctae in the HBV at 6 and 24 hpi of Ctrl (grey) or macrophage-ablated (Mtz; black) larvae infected with type I *Toxoplasma*-GFP. Mean ± SEM shown. Pooled data from 3 independent experiments with at least 7 larvae per condition per experiment. Significance calculated using 2-way ANOVA (repeated measures) with Sidak's multiple comparisons test, *, $p\leq0.05$, **, $p\leq0.01$. (F) Quantification of input dose of type II (semi-closed circle, left graph) or type III (closed circle, right graph) *Toxoplasma*-GFP in Ctrl (grey) or macrophage-ablated (Mtz; black) larvae using particle analysis of

Disease Models & Mechanisms • Supplementary information

infected HBV images obtained by fluorescent stereomicroscopy at 0 hpi. Mean ± SEM shown. Significance calculated using unpaired t-test, ns, $p>0.05$. (G) Quantification of parasite burden in the HBV of Ctrl (grey) or macrophage-ablated (Mtz; black) larvae infected with type II (semi-closed circle, left graph) or III (closed circle, right graph) at 24 hpi. Mean ± SEM shown. Showing 1 representative experiment of 3 with at least 10 larvae per condition per experiment. Significance calculated using unpaired t-test, **, $p\leq0.01$, ***, $p\leq0.001$.

Disease Models & Mechanisms • Supplementary information

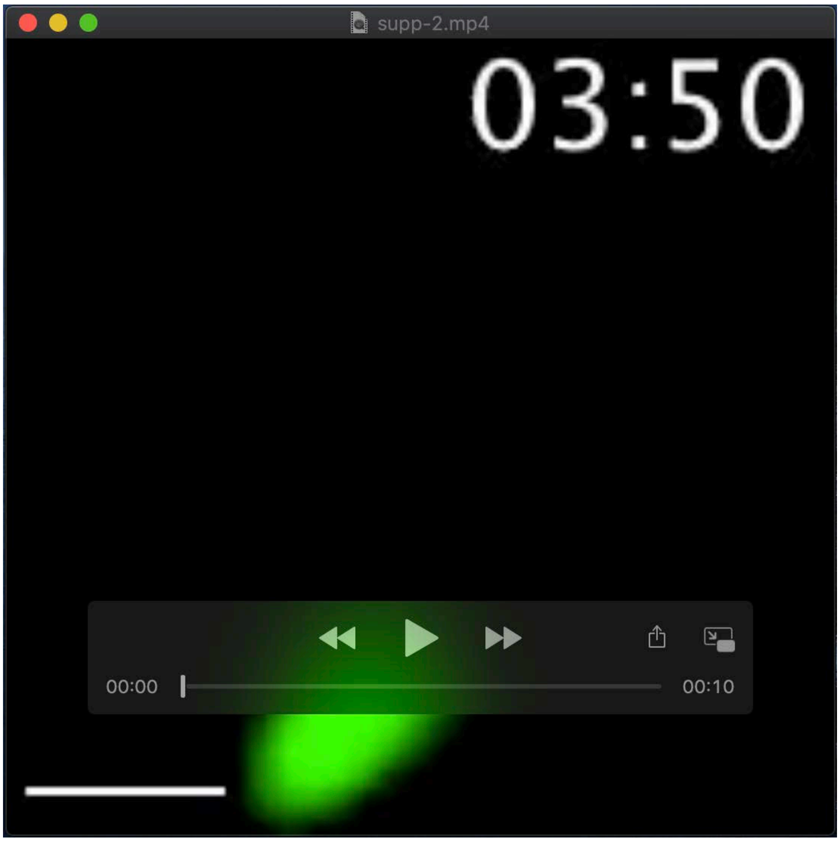

Movie 1. *In vivo* replication of *Toxoplasma gondii*, related to Figure S1B.

In vivo fluorescent widefield imaging of zebrafish larvae injected with type I *Toxoplasma*-GFP (green). First frame at 3 h 50 minutes post-infection (mpi) followed by frames taken at 10 minute intervals until 8 h 30 mpi. Showing a single Z-plane from 60 taken at 2 μm optical section. Scale bar, 5 μm.

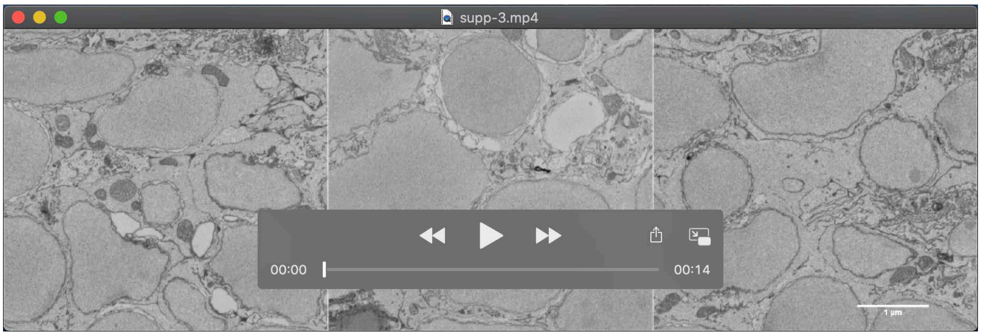

Movie 2. 3D CLEM of *Toxoplasma gondii* replication in the zebrafish hindbrain, related to Figure 2A. SBF SEM of tachyzoites in the HBV of larvae injected with type I *Toxoplasma*-GFP at 6 hpi. Representative examples of single (left), replicating (middle) and replicated doublet (right) *Toxoplasma* from a total of 36 found in zebrafish cells and imaged in their full volume to accurately determine their stage (see Fig. 2A and Fig. S2). 112 consecutive 50 nm SBF SEM slices of a different subregion of a section for each example. Scale bar, 1 µm.

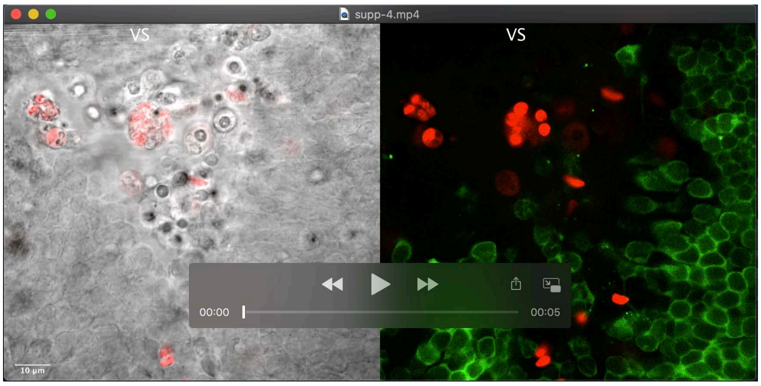

Movie 3. *Toxoplasma* tachyzoites reside within neurons, related to Figure 2B. *In vivo* confocal microscopy imaging of live 3 dpf Tg*(elavl3:GCaMP6s)*^{jf4} larvae (neurons marked in green) infected in the HBV with type I *Toxoplasma*-Tomato (red) at 4 hpi. A Z-stack of 33 Z-slices (Z=0.43 μm) is shown at 6 fps showing brightfield (grey) and *Toxoplasma* (red) composite (left) or *Toxoplasma* (red) and neurons (green) composite (right). Ventricular surface labeled as VS. The five tachyzoites shown in Fig. 2B are labeled with white arrowheads. Scale bar, 10 μm.

Disease Models & Mechanisms • Supplementary information

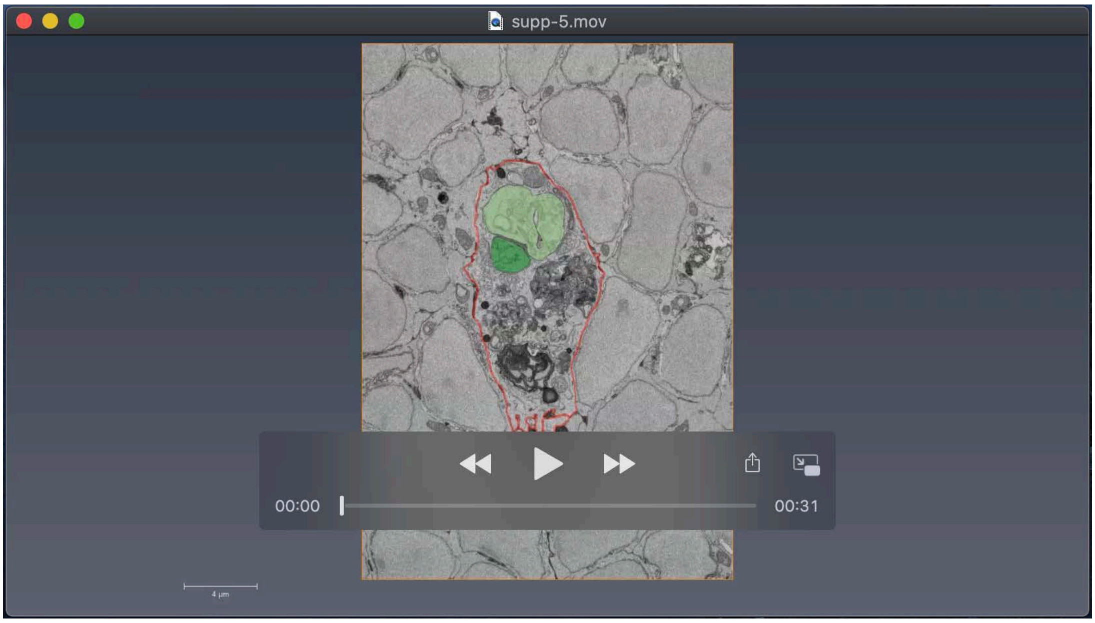

Movie 4. 3D CLEM of replicative *Toxoplasma gondii* inside a zebrafish macrophage, related to Figure S5B. SBF SEM of intraphagocytic parasite replication in the HBV of *mpeg1*:G/U:mCherry larvae infected with type I *Toxoplasma*-GFP at 6 hpi. 373 consecutive 50 nm SBF SEM slices in which the replicating *Toxoplasma* (green and light green) and plasma membrane of the macrophage (red) were manually segmented. A surface was generated to build a 3D model of the 2 segmented *Toxoplasma* in Amira software. Scale bar, as indicated.

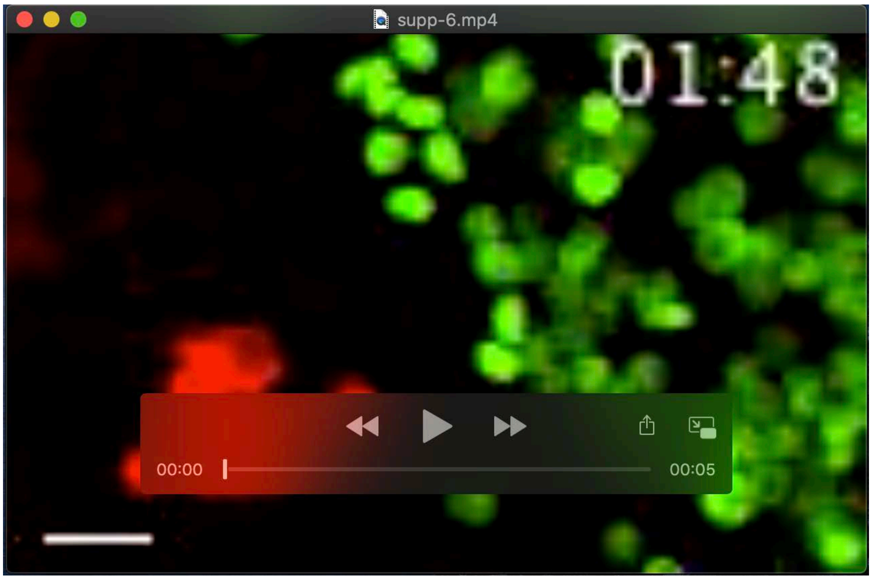

Movie 5. Phagocytosis of *Toxoplasma gondii* by a macrophage, related to Figure 5A. *In vivo* confocal imaging of *mpeg1*:G/U:mCherry larvae harboring red macrophages injected with type I *Toxoplasma*-GFP (green). First frame at 1 h 48 mpi followed by frames taken at 8 minute intervals until 3 h 48 mpi. Showing a maximum projection of 24 Z-slices from 60 taken at 2 μm optical sections. Scale bar, 10 μm.

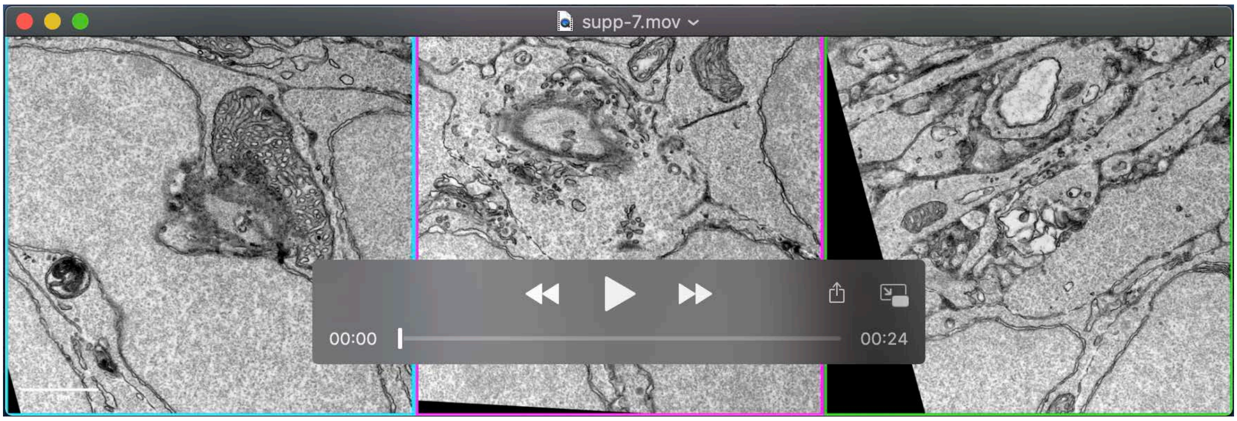

Movie 6. Host cell-intrinsic response disrupts *Toxoplasma* parasitophorous vacuoles in zebrafish brain cells *in vivo*, related to Figure 6. ssTEM of parasite replication in brain cells in the HBV of *mpeg1*:G/U:mCherry larvae infected with type I *Toxoplasma*-GFP at 6 hpi. Movie shows 71 consecutive 70 nm sections imaged by ssTEM at 6800x magnification at 3 fps. *Toxoplasma* tachyzoites were imaged in their full volume to accurately assess the continuity of the PV. Color boxes (left- cyan box, middle- magenta box, right- green box) match the ones in Fig. 6. Scale bar, 1 μm.

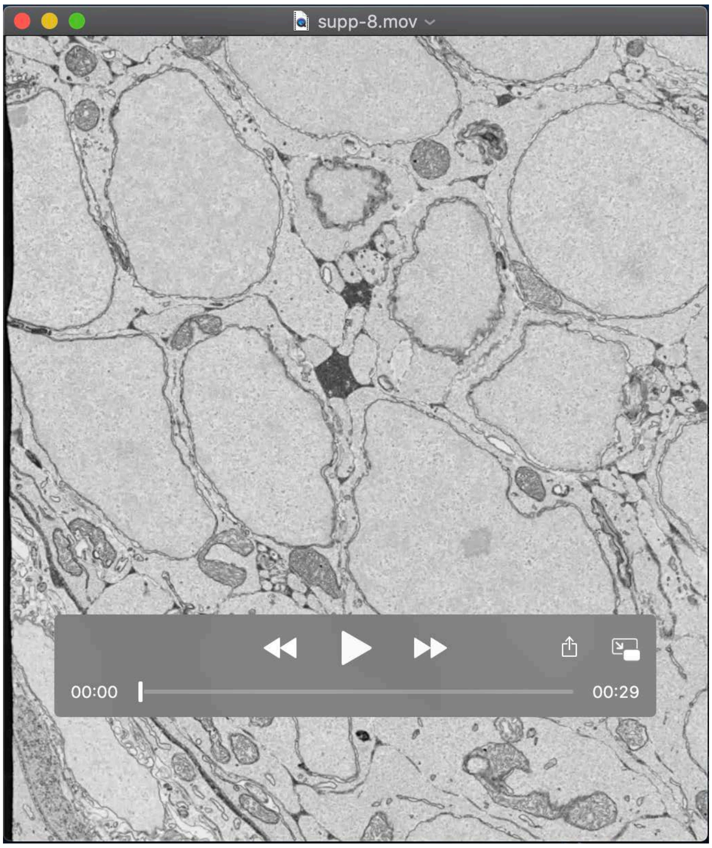

Movie 7. Host cell-intrinsic response disrupts *Toxoplasma* parasitophorous vacuoles in zebrafish macrophages *in vivo*, related to Figure 7*.* FIB SEM of two parasites inside a macrophage in the HBV of *mpeg1:G/U*:mCherry larvae infected with type I *Toxoplasma*-GFP at 6 hpi. The data was binned to 20 nm^3 to fit within the size constraints. Movie shows 860 consecutive slices through the YZ axis, as shown in Fig. 7. Scale bar, 5 μm (top panels) and 1 μm (lower panels). Field of view of 16 x 19 μm in XY.

Disease Models & Mechanisms • Supplementary information

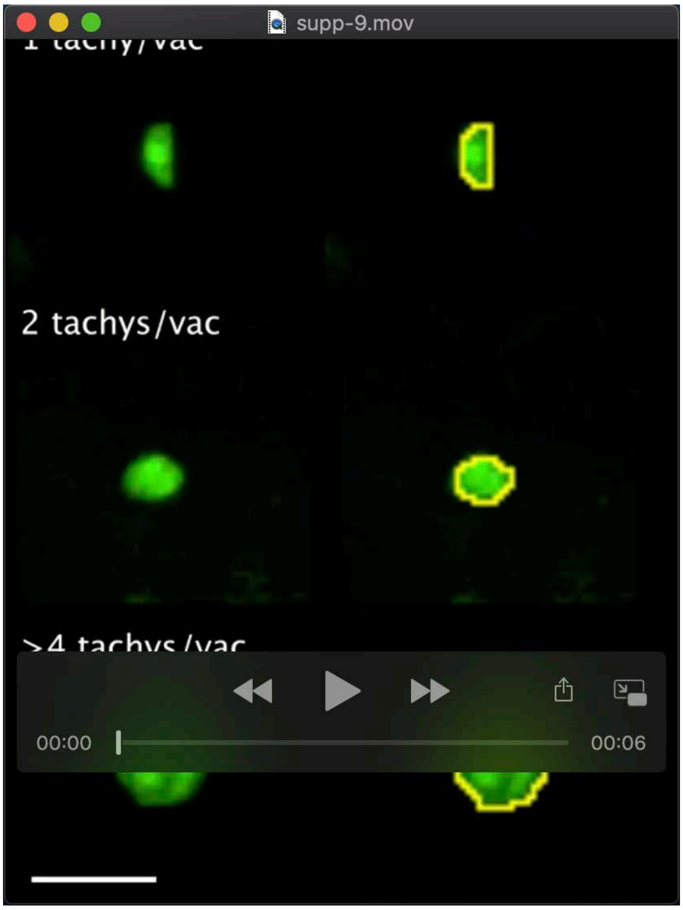

Movie 8. 3D visualization of *Toxoplasma gondii* replication in the zebrafish hindbrain by confocal microscopy for pixel volume analysis. Showing representative 3D reconstruction of confocal images of GFP-positive replicating tachyzoites in fixed zebrafish larvae infected with type I *Toxoplasma*-GFP (green). Volumes were categorized into 1 tachyzoite/vacuole (<50 pix^3, top), 2 tachyzoites/vacuole (50<100 pix^3, middle) or >4 tachyzoites/vacuole (>100 pix^3, bottom). Outline of GFP-positive punctae volumes measured is in yellow (left). Scale bar, 10 µm.

Disease Models & Mechanisms • Supplementary information
